# New Synthetic Strategies Toward DFO*: Enhanced Yield and Purity of a Key Chelator for ^89^Zr Chemistry

**DOI:** 10.3390/ph19060813

**Published:** 2026-05-22

**Authors:** Nils F. Baier, Minqian Miao, Ralf Schirrmacher, Björn Wängler, Gert Fricker, Carmen Wängler

**Affiliations:** 1 Biomedical Chemistry, Clinic of Radiology and Nuclear Medicine, Medical Faculty Mannheim, Heidelberg University, Theodor-Kutzer-Ufer 1-3, 68167 Mannheim, Germany; nils.baier@medma.uni-heidelberg.de (N.F.B.);; 2 Research Campus M^2^OLIE, Medical Faculty Mannheim, Heidelberg University, Theodor-Kutzer-Ufer 1-3, 68167 Mannheim, Germany; 3 Division of Oncological Imaging, Department of Oncology, University of Alberta, Edmonton, AB T6G 1Z2, Canada; 4 Molecular Imaging and Radiochemistry, Clinic of Radiology and Nuclear Medicine, Medical Faculty Mannheim, Heidelberg University, Theodor-Kutzer-Ufer 1-3, 68167 Mannheim, Germany; 5 Mannheim Institute for Intelligent Systems in Medicine (MIISM), Medical Faculty Mannheim, Heidelberg University, 68167 Mannheim, Germany; 6 Institute of Pharmacy and Molecular Biotechnology, Heidelberg University, 69120 Heidelberg, Germany

**Keywords:** DFO, DFO*, hydroxamate chelators, acid lability, ^89^Zr

## Abstract

**Background**: Zirconium-89 (^89^Zr) is a key PET radionuclide and the limited in vivo stability of its clinically used ^89^Zr-DFO complexes has driven the pursuit of improved chelator architectures. Among these, DFO* has attracted particular attention due to its exceptional complex stability with ^89^Zr^4+^ and favorable pharmacokinetics of the corresponding bioconjugates in vivo. Despite these advantages, DFO*’s broader application has been hampered by significant synthetic challenges, primarily arising from its pronounced acid sensitivity. **Methods**: Here, we present a systematic investigation of the acid lability of DFO and DFO*-derived systems, revealing substantial degradation under acidic conditions being commonly applied during preparation and purification. These findings highlight critical limitations of conventional synthetic and purification protocols. To address this, we developed two complementary synthetic routes that consistently avoid fragmentation-inducing conditions. **Results**: THP/Boc- and TBDPS/Fmoc-based routes provide robust five- and six-step syntheses of DFO*, affording overall yields of 11% and 13%/6.1% and high purity (≥98%) without detectable degradation. **Conclusions**: By systematically investigating the acid sensitivity of DFO/DFO*-based chelators and providing practical synthetic solutions, this work enables reliable access to DFO* and advances its application in ^89^Zr radiopharmaceutical development.

## 1. Introduction

Zirconium-89 (^89^Zr) has emerged as a key radionuclide for positron emission tomography (PET) imaging in nuclear medicine. Its comparatively long physical half-life (3.3 days) distinguishes ^89^Zr from many other positron emitters and enables the visualization of slow pharmacokinetic and biological processes over extended timeframes. Consequently, ^89^Zr is particularly well suited for imaging the distribution of large biomolecules, such as monoclonal antibodies, whose in vivo pharmacokinetics and highly specific target accumulation occur on timescales of several days [1,2]. The favorable match between the biological half-life of antibodies and the physical half-life of ^89^Zr has established ^89^Zr-based immuno-PET as an important tool in preclinical and clinical PET imaging [2,3].

Target-specific bioactive molecules are, in general, labeled with ^89^Zr through chelator-mediated complexation. These chelators ideally form kinetically inert and thermodynamically stable complexes with the metal ion to prevent release from the biological carrier. In the clinical setting, desferrioxamine B (DFO) remains the only chelator used for ^89^Zr complexation; however, the resulting [^89^Zr]Zr-DFO complex is known to exhibit limited in vivo stability. Partial dissociation of ^89^Zr^4+^ from [^89^Zr]Zr-DFO can occur over time, leading to the release of free zirconium ions [4,5]. This instability poses a significant problem for safety and image quality. Free ^89^Zr^4+^ exhibits a strong affinity for hydroxylapatite and therefore accumulates in bone mineral [6]. Such nonspecific distribution and accumulation not only compromise PET image quality by resulting in increased background but also result in an unnecessary radiation burden to the healthy tissues, particularly the bone marrow and thus hematopoietic stem cells [7,8]. Consequently, the development of alternative chelators that provide improved stability of ^89^Zr complexes has been a major focus of research in recent years [7].

Among these newly developed chelators, the octadentate DFO analog DFO* [9] has attracted particular attention. Studies have demonstrated that DFO* forms exceptionally stable complexes with ^89^Zr^4+^ [10,11,12] and, when introduced into antibodies, exhibits favorable pharmacokinetics compared with other advanced chelators including HOPO derivatives [1]. These properties underscore the strong potential of DFO* for clinical application in ^89^Zr-based radiopharmaceuticals and suggest that it can address key limitations associated with conventional [^89^Zr]Zr-DFO systems.

Despite its promising radiochemical and biological performance, a broader application of DFO* has so far been limited. One probable reason is the considerable synthetic challenge associated with this molecule, which exhibits pronounced instability towards acidic conditions. To date, the literature has exclusively described the base lability of hydroxamates [13], whereas reports on their potential acid lability are lacking. The pronounced acid sensitivity of DFO*, even under mildly acidic conditions, limits the applicability of conventional acid-labile protecting group strategies. Moreover, standard HPLC purification under acidic conditions compromises yield and purity, restricting access to DFO* in high purity and adequate quantities.

Accordingly, there is a need for a systematic evaluation of the acid sensitivity of DFO-derived systems, together with a synthetic approach to DFO* that avoids fragmenting conditions during synthesis and purification and enables efficient access to this chelator in high yield and purity. In this work, we close this gap by systematically investigating the acid lability of DFO and DFO* and by presenting two newly developed synthetic routes for DFO* which avoid exposure of the molecule to deteriorating conditions.

Collectively, these strategies provide robust access to DFO* in high yield and purity, supporting its broader use in the development and production of stable ^89^Zr radiopharmaceuticals.

## 2. Results and Discussion

### 2.1. Systematic Investigation of the Acid Sensitivity of DFO and DFO* Towards Different Acidic Conditions

**Scheme 1:**
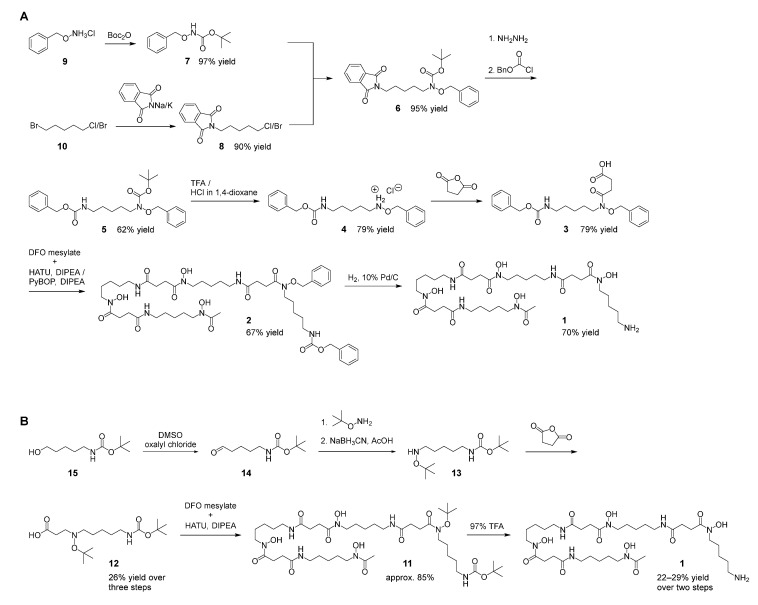
Schematic representation of the two established synthetic routes to DFO* (**1**), employing either reductively cleavable (**A**) or acid-labile (**B**) protecting groups.

To date, two distinct synthetic routes to DFO* (**1**) have been reported. In one comparatively complex approach, the DFO* is obtained over eight steps employing reductively cleavable benzyl (Bn) and benzyloxycarbonyl (Cbz) protecting groups (Scheme 1A). The final product is isolated either by precipitation or by HPLC purification, affording overall yields ranging from 7.9% [9,14] to 15.5% [11] over all steps. In addition to the complex synthesis, the final deprotection of the Bn and the Cbz protecting groups by hydration can result in a partial reduction in the hydroxamates to amides which are rather difficult to separate from the intended product.

Thus, a second synthetic strategy was developed recently that enables access to **1** in only five steps using acid-labile protecting groups (Scheme 1B) [15]. In this approach, **1** is released in the final step by incubation of the *tert*-butyl (*^t^*Bu) and *tert*-butoxycarbonyl (Boc) protected intermediate **11** with 97% TFA, followed by precipitation and small-scale cartridge purification in milligram quantities. Over the entire pathway, **1** is obtained in final purities of 92–98% with an overall yield of 7.5% [15]. This route is streamlined by advancing the first two and the fourth step without purification, yielding a more concise synthesis despite lower overall yields. However, this synthetic route is poorly scalable due to the final cartridge purification. Further, the final deprotection step from **11** to **1** results in substantial losses in product yield due to the acid-sensitivity of the tetrahydroxamate system (Scheme 1B).

In our experience, inefficient *tert*-butyl deprotection of **11** leads to extensive side-product formation (Figure S25), resulting in intricate HPLC purification and markedly reduced isolated yields. Analysis of the individual compounds by LC-MS revealed that most of these side products correspond to fragmented species, predominantly arising from cleavage of the hydroxamic acid groups, producing the corresponding fragments (hydroxamates and carboxylic acids). Mechanistically, a protonation of the carbonyl group takes place first, followed by a nucleophilic attack of water on the carbonyl carbon, which has become electrophilic due to the protonation; this is followed by proton transfer and protonation of the amide nitrogen, and finally the cleavage of the bond of the parent hydroxamic acid to form the carboxylic acid and hydroxylamine. As well, the purification of **1** by HPLC using 0.1% TFA as an additive in the mobile phase fails to afford the product in high purity. Notably, analysis of the HPLC fractions immediately after separation indicated a chemically pure and intact product. These observations suggest a pronounced acid lability of DFO* and its derivatives even under dilute acidic conditions, which, to the best of our knowledge, has not been reported previously. This prompted a systematic investigation of the acid sensitivity to guide the development of an improved synthetic route to **1**.

**Figure 1:**
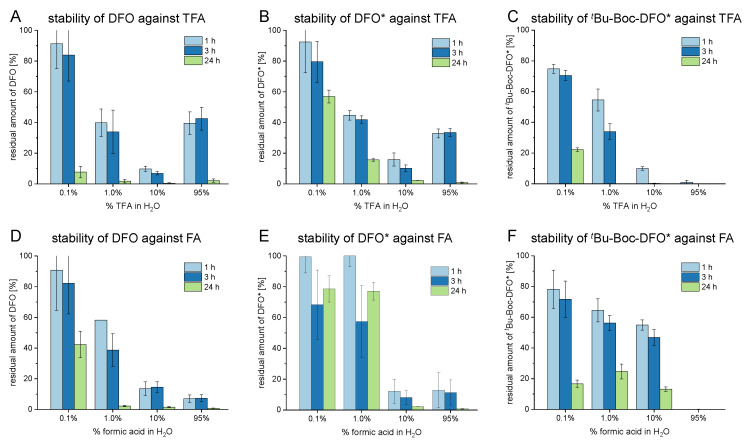
Summary of the acid stability evaluations of 1 mM DFO mesylate (**A**,**D**), DFO* (**B**,**E**), and intermediate *^t^*Bu-Boc-DFO* (**C**,**F**) upon exposure to TFA and FA at varying acid concentrations of 0.1%, 1%, 10% and 95% after incubation times of 1, 3, and 24 h.

To this end, we first examined whether DFO and DFO* undergo degradation under typical HPLC purification conditions. Common additives to RP-18 HPLC eluents (water and acetonitrile) include 0.1% trifluoroacetic acid (TFA) or 0.1% formic acid (FA). Accordingly, pure DFO mesylate and DFO* were incubated at room temperature for 1, 3, and 24 h in water containing 0.1% TFA or FA. In both cases, considerable decomposition was observed by analytical HPLC; however, both compounds exhibited higher susceptibility toward TFA compared to FA (Figure 1A vs. Figure 1D and Figure 1B vs. Figure 1E). Interestingly, DFO* displayed increased acid stability in comparison to DFO under these conditions (Figure 1B vs. Figure 1A and Figure 1E vs. Figure 1D). For comparison, intermediate **11** was also evaluated under identical conditions to analyze the kinetics of *^t^*Bu and Boc protecting group cleavage and formation of **1**.

When the concentrations of TFA and FA were increased from 0.1% to 1%, 10%, and 95% in water, decomposition was generally accelerated with increasing acid concentration (Figure 1A,B,D,E). The only exception from this was observed for the incubation of DFO and DFO* with 95% TFA, which resulted in lower degradation rates than those observed in 10% TFA (Figure 1A,B). This effect may arise from insufficient water content to enable efficient hydroxamic acid cleavage in highly concentrated TFA; however, substantial fragmentation (~60%) was nevertheless observed in 95% TFA within 1 h (Figure 1A,B). These findings explain the unexpectedly low yields described for the final step of the synthesis route of DFO* relying on acid-labile *^t^*Bu and Boc protecting groups (Scheme 1B) [15]. Building on these observations, the acid lability of the *tert*-butyl- and Boc-protected precursor **11** was systematically investigated as well. Upon exposure to either TFA or FA, rapid consumption of **11** (Figure 1C,F), accompanied by formation of **1**, was observed. Notably, deprotection of **11**—especially using 95% TFA as deprotecting agent—proceeded more rapidly than degradation of **1** (Figure 1C vs. Figure 1B), which explains why **1** can be isolated in relevant amounts despite its intrinsic sensitivity to the highly acidic deprotection conditions of 97% TFA [15].

**Figure 2:**
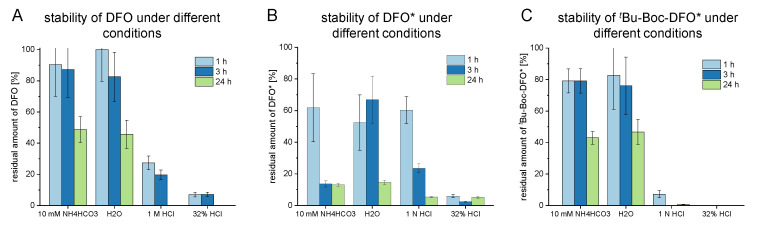
Summary of the acid stability evaluations of 1 mM DFO mesylate (**A**), DFO* (**B**), and *^t^*Bu-Boc-DFO* (**C**) upon exposure to 10 mM ammonium carbonate solution, pure H_2_O, 1 M and 32% HCl after incubation times of 1, 3, and 24 h.

Finally, we intended to determine whether DFO and DFO* (and its precursor **11**) might exhibit a higher stability toward acids other than TFA and FA. To this end, the experiments described above were repeated using 10 mM ammonium carbonate solution, pure H_2_O, and aqueous HCl (1 M and 32%). Notably, substantial degradation of DFO, DFO*, and **11** was already observed in pure water, which rationalizes the pronounced decomposition detected under all further conditions tested (Figure 2).

After establishing the acid lability of DFO and DFO*, we sought to develop alternative synthetic routes to DFO* that require fewer steps than the reductively cleavable protecting group-based approach and afford the final product in higher yield and purity than the previously employed strategy based on acid-labile groups. To this end, two complementary approaches, A and B, were pursued.

### 2.2. Synthetic Strategy A: Optimization of the Acid-Labile Protecting Group-Based Synthesis Route to DFO*

**Figure 3:**
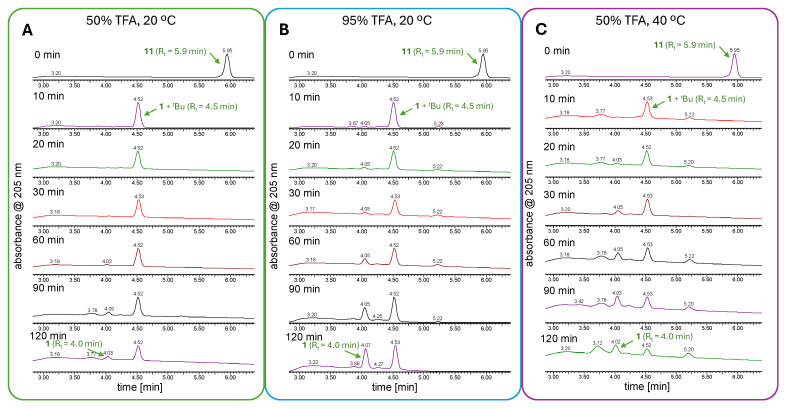
Compilation of analytical HPLC chromatograms monitoring the stepwise deprotection of the *tert*-butyl and Boc protecting groups during the conversion of **11** to **1** under different reaction conditions. In all experiments, ultrasound was applied to enhance reaction efficiency.

As discussed above, the synthetic route to compound **1** via the acid-labile protecting group strategy proceeds with high efficiency up to the final step. However, this last step results in a substantial loss in overall yield. A marked difference in protecting group lability was observed: cleavage of the *tert*-butyl group necessitates high TFA concentrations and extended reaction times, while the Boc group is removed rapidly and completely within 10 min under comparatively mild acidic conditions (Figure 3). This observation is consistent with the literature reports [16].

Treatment of **11** with 50% TFA at ambient temperature resulted in quantitative formation of the *^t^*Bu-protected intermediate within 10 min, while formation of **1** was slow and observed only in minor amounts within 120 min (Figure 3A). Treatment with 95% TFA at ambient temperature likewise led to quantitative formation of the *^t^*Bu-protected intermediate within 10 min; however, after 120 min, significant amounts of **1** were detected alongside fragmentation products and an overall decrease in detectable material (Figure 3B). Treatment with 50% TFA at elevated temperature (40 °C) led, in addition to the formation of the *^t^*Bu-protected intermediate, to substantial by-product formation already after 10 min (Figure 3C). Notably, the formation of side products (t_R_ < 4.0 min) was observed under all conditions, accompanied by a general decrease in total detectable substance.

We therefore considered whether the synthesis of DFO* could be improved by replacing the *^t^*Bu moiety with an alternative acid-labile protecting group that allows for significantly faster deprotection, circumventing the prolonged exposure of the acid-labile tetrahydroxamate system to acidic conditions.

A review of the protecting group literature identified the tetrahydropyranyl (THP) group as a promising replacement for the *tert*-butyl group, owing to its stability under neutral to basic conditions and its efficient cleavage in aqueous media at pH ≤ 4 [16]. This profile renders THP well suited for minimizing hydroxamate degradation during the final deprotection to **1**. Accordingly, we sought to develop a novel synthetic route to DFO* that employs an *O*-THP protecting group in place of the commonly used *O*-*^t^*Bu group, thereby enabling the avoidance of harsh acidic deprotection conditions that lead to the fragmentation of **1**.

**Scheme 2:**
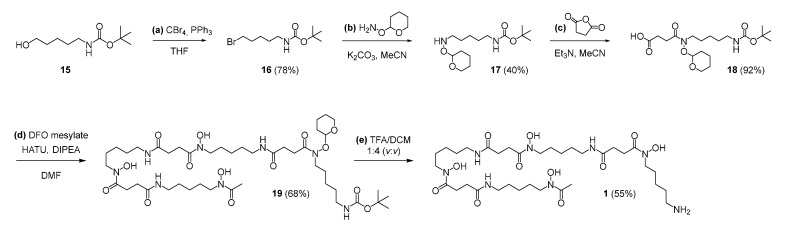
Schematic representation of the synthetic route A to DFO*, being based on acid-sensitive Boc and THP protecting groups. Reaction conditions: (**a**) PPh_3_ (1.5 eq.), CBr_4_ (1.6 eq.), THF, 0 °C → ambient temperature, o.n., 78% yield; (**b**) K_2_CO_3_ (4 eq.), THP-hydroxylamine (2 eq.), MeCN, 60 °C, o.n., 40% yield; (**c**) NEt_3_ (2 eq.), succinic anhydride (1.5 eq.), MeCN, ambient temperature, o.n., 92% yield; (**d**) HATU (1 eq.), DIPEA (2 eq.), DMF, ambient temperature, 15 min, DFO mesylate (0.66 eq.), 40 °C, 48 h, 68% yield; (**e**) TFA:DCM (1:4, *v*:*v*), 20 °C, 5 min, 55% yield.

The developed synthesis approach A (Scheme 2) started from *N*-Boc-5-aminopentan-1-ol (**15**), which was converted into the corresponding bromide (**16**) using CBr_4_ and triphenylphosphine. Subsequent reaction of **16** with *O*-(tetrahydro-2H-pyran-2-yl)hydroxylamine afforded the *N*-Boc- and *NO*-THP-protected intermediate **17,** which was reacted with succinic anhydride to yield the fully protected building block **18**. The carboxylic acid of **18** was in the following activated to react with this building block with DFO mesylate to give the THP- and Boc-protected precursor of DFO*, **19**. The final deprotection of both the Boc and THP groups was achieved under mildly acidic conditions (20% TFA in DCM) within only 5 min, followed by immediate removal of the acid, purification of product **1** via preparative HPLC and immediate lyophilization of product fractions. This remarkably high reaction efficiency of the final deprotection step was achieved by conducting the reaction under ultrasound assistance. Ultrasound has repeatedly been shown to enhance reaction efficiencies and thereby accelerate transformations [17,18]. Although this effect was not systematically investigated for the deprotection of **19** to **1** in the present study, small-scale preliminary experiments revealed that the omission of ultrasound necessitated prolonged reaction times and thus resulted in the formation of significantly higher amounts of byproducts.

All intermediates and the final product were obtained in low three-digit mg quantities, purities of ≥95% and in good to excellent yields for the individual steps, ranging from 40 to 92%, corresponding to an overall yield of 11% over all five reaction steps. Although purification of the final product **1** by HPLC was also performed along this synthetic route—analogous to the previously reported pathway via intermediate **11**—the purification proved to be considerably more efficient than using the conventional protecting group strategy. This improvement can be attributed to the mild deprotection conditions applicable here, resulting in the formation of virtually no side products, which otherwise complicate purification and contribute to substantial yield losses. Alternatively, the purification of **1** can also be accomplished by flash chromatography, substantially reducing purification time.

### 2.3. Synthetic Strategy B: Alternative Reaction Route to DFO*, Based on TBAF-Labile Protecting Group Chemistry

In parallel to the outlined synthetic route A described above, an alternative approach was devised being designed to avoid acidic conditions altogether. This strategy was based on the use of protecting groups being labile against treatment with TBAF, which is a very mild cleavage agent. An additional objective was to eliminate the need for HPLC purification of the final product, thereby, in principle, enabling the preparation of DFO* in gram scales.

**Scheme 3:**
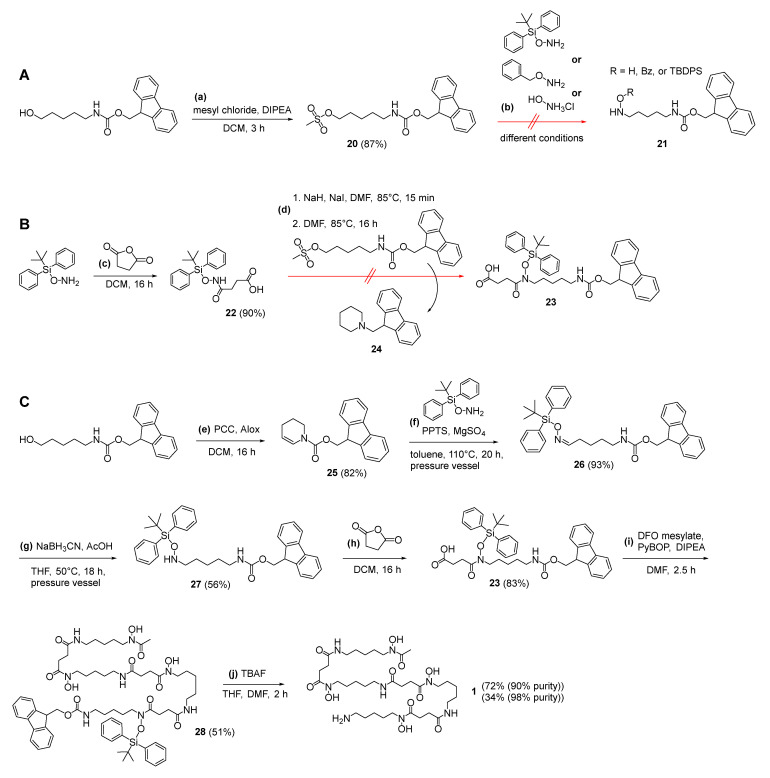
Schematic representation of the synthetic route to DFO* based on the use of TBAF-sensitive Fmoc and TBDPS protecting groups. Panels (**A**,**B**) show the non-successful synthesis routes, while panel (**C**) depicts the successful reaction pathway to **1**. Reaction conditions: (**a**) mesyl chloride (1.2 eq.), DIPEA (2.1 eq.), DCM, 0 °C, 3 h, 87% yield; (**b**) different conditions—no product; (**c**) succinic anhydride (3.0 eq.), DCM, ambient temperature, 2.5 h, 90% yield; (**d**) NaH, NaI, DMF, 85 °C, 15 min, followed by **20**, DMF, 85 °C, 16 h—no product; (**e**) PCC (1.5 eq.), Alox, DCM, 20 °C, 16 h, 82% yield; (**f**) *O*-(*tert*-butyldiphenylsilyl)hydroxylamine (1.3 eq.), PPTS (0.036 eq.), toluene, 110 °C, 20 h, 93% yield; (**g**) NaBH_3_CN (10 eq.), acetic acid (2.5 eq.), THF, 50 °C, 18 h, 56% yield; (**h**) succinic anhydride (3.2 eq.), DCM, 20 °C, 16 h, 83% yield; (**i**) PyBOP (1.17 eq.), DIPEA (1.67 eq.), 20 °C, 1 h, DFO mesylate (0.83 eq.) 20 °C, 1.5 h, 51% yield; (**j**) TBAF (12 eq.), DMF:THF 5:1 (*v*:*v*), 20 °C, 1 h, 79% yield.

A combination of 9-fluorenylmethoxycarbonyl (Fmoc) and *tert*-butyldiphenylsilyl (TBDPS) protecting groups appeared particularly advantageous, as both were expected to be cleavable under very mild conditions. For this purpose, a TBAF-mediated global deprotection was envisioned (Scheme 3), leveraging the stability of the DFO* hydroxamate scaffold. This approach involved assembly of Fmoc- and TBDPS-protected building block **23**, conjugation to DFO mesylate to form **28**, and final deprotection of this precursor to yield DFO*.

For the preparation of building block **23**, a viable synthetic route had to be established first. The initial two synthetic approaches toward this agent (Scheme 3A,B) did not afford the desired compound. In the first attempt (Scheme 3A), (9*H*-fluoren-9-yl)methyl-(5-hydroxypentyl)carbamate was converted into the corresponding mesylate **20** using mesyl chloride, with the intention of introducing a protected hydroxylamine nucleophile in a subsequent step to afford the respective intermediate **21**. This would then have been reacted with succinic anhydride to provide the target building block **23**. However, mesylate **20** exhibited remarkably low reactivity, and no conversion to **21** was observed with any of the hydroxylamines examined, including *O*-TBDPS-, *O*-benzyl-, or unprotected hydroxylamine. Thus, extensive variation in reaction parameters was undertaken in an effort to induce conversion. For this purpose, mesylate **20** was reacted with *O*-TBDPS hydroxylamine in MeCN in the presence of K_2_CO_3_, as well as in DCM with DIPEA, both with and without LiH activation, at ambient temperature and at elevated temperatures of up to 80 °C. These conditions led either to mesylate hydrolysis or exclusive formation of the intramolecular product **24**, with no conversion to **23**. To rule out steric hindrance imposed by the bulky TBDPS protecting group, mesylate **20** was also treated with unprotected hydroxylamine under a wide range of reaction conditions. These included reactions of **20** with hydroxylamine in DCM or MeOH with DIPEA (with and without KI), in DMF or MeCN/DMF using K_2_CO_3_ or NaHCO_3_, and in DMF, THF, or MeOH in the presence of LiH, DIPEA, or in the absence of any base. Across all conditions examined, reactions resulted in mesylate hydrolysis, formation of by-product **24**, or decomposition to non-characterizable products, with no productive conversion observed. An alternative synthetic strategy was therefore pursued (Scheme 3B), involving conversion of TBDPS-protected hydroxylamine to acid **22** and subsequent reaction with mesylate **20**. Although the formation of **22** proceeded cleanly, conjugation to **20** again failed to deliver the target compound **23**. Instead, only the intramolecular byproduct **24** could again be isolated, explaining the general lack of reactivity observed for the mesylate toward various nucleophilic building blocks observed given that the intramolecular reaction proceeds preferentially.

Consequently, a third strategy was explored (Scheme 3C), which avoided activation of the hydroxyl group of (9*H*-fluoren-9-yl)methyl (5-hydroxypentyl)carbamate, but instead relied on its oxidation to **25**, followed by its pyridinium p-toluene sulfonate (PPTS)-catalyzed Schiff base reaction with the TBDPS-protected hydroxylamine to give both oxime configuration isomers of **26** with in an *E*/*Z* ratio of 0.61/0.39. The identification of the configuration isomers was carried out with NOE experiments with selective excitation of the aldoxime protons at the chemical shifts of 7.75 and 7.08 ppm. Due to the sterical hindrance exerted by the TBDPS protection group, the formation of the *E* isomer is favored. This latter reaction was carried out in a pressure vessel to increase reaction efficiency. Subsequent reduction of **26** using NaBH_3_CN and acetic acid gave amine **27**. The transformation to **27** required careful optimization to prevent partial or complete loss of protecting groups and to ensure full conversion. Reduction with NaBH_3_CN in THF in the presence of acetic acid led to product formation at both ambient temperature and 85 °C; however, even after 16 h, residual starting material was consistently detected. Complete conversion under otherwise identical conditions was achieved only upon conducting the reaction in a sealed pressure vessel. This proved essential, as the separation of the product from unreacted starting material proved to be challenging. Alternative reductive agents—including NaBH_4_ in THF with AcOH as additive, BH_3_ in THF in the presence of HCl, LiAlH_4_ in THF, and catalytic hydrogenation (H_2_, Pt/C)—resulted in the partial or complete loss of protecting groups and were therefore not pursued further. Consequently, the reduction with NaBH_3_CN in THF using AcOH as the additive at 85 °C in a pressure vessel was identified as the optimal protocol, affording the desired product **27** in 56% yield after workup. Subsequent reaction of **27** with succinic anhydride furnished the TBDPS- and Fmoc-protected building block **23** in 83% yield and 35% overall yield over four steps. Furthermore, **23** was subsequently reacted with DFO mesylate to give the TBDPS- and Fmoc-protected DFO* precursor **28** in 51% yield. Complete deprotection of **28** to afford DFO* was ultimately achieved under very mild conditions using TBAF, as intended, without any observable fragmentation of the hydroxamate backbone. To circumvent HPLC purification, a recrystallization protocol from boiling 2-propanol was established, providing **1** in 72% yield and 90% purity. This proved to be sufficient for further functionalization reactions, as we could not detect any adverse effects from the 10% impurities on the subsequent reactions, although we were unable to identify the impurity. Nevertheless, purity can be further increased by an additional purification step using flash chromatography to 98% while reducing the yield to 34%.

### 2.4. Comparison of Both Synthesis Routes

A final assessment as to which of the two developed routes to DFO*-amine is superior is not straightforward; rather, the decision depends on the requirements and practical preferences. Both strategies afford the target compound **1** in comparable overall yields (11% using acid-labile THP and Boc protecting groups and 13%/6.1% using TBAF-labile TBDPS and Fmoc protecting groups) and deliver highly pure material in each case (≥98%) in five or six reaction steps. Moreover, both approaches enable access to the product without noticeable fragmentation. This represents a clear advantage over previously reported synthetic routes, which—due to the acid lability of the hydroxamate backbone and the requirement for harsh acidic cleavage conditions of *^t^*Bu and Boc protecting groups in the final step—provided substantially lower yields or, in the case of the reductively cleavable Bn/Cbz protecting group strategy, required significantly greater synthetic effort involving eight reaction steps furthermore bearing the significant risk of the over-reduction in the product.

The two complementary strategies developed herein differ in reaction step count and operational handling, yet both represent a clear advancement over previously reported protocols in terms of robustness, scalability, and product purity and accessibility. The acid-labile THP/Boc-based route (synthesis route A) proceeds in five steps and enables access to the target compound in high purity via either preparative HPLC or flash chromatography. Notably, HPLC purification under acidic conditions directly delivers the protonated form of the product, which exhibits markedly improved solubility not only in DMSO but also in methanol and DMF. This feature constitutes a significant practical advantage for downstream transformations, including further functionalization and bioconjugation. While preparative HPLC inherently limits the isolated quantities to the low three-digit milligram scale and requires careful handling of product solutions to avoid decomposition, switching to flash chromatography enables straightforward scale-up to larger quantities in the gram scale, albeit at the expense of reduced solubility due to a lower degree of protonation. In contrast, the TBAF-labile TBDPS/Fmoc-based approach (synthesis route B) comprises six steps but entirely avoids aqueous conditions, thereby effectively suppressing hydroxamate fragmentation of both intermediates and the final product. Importantly, this route allows for product isolation by precipitation and recrystallization, or flash chromatography, eliminating the need for preparative HPLC and facilitating access to gram-scale quantities of the product. Accordingly, for the deprotection reaction converting **28** to **1**, we achieved an isolated yield of 33% for **1** on a scale of up to 1.6 g of **28** using flash chromatography for purification of the product. In contrast, purification by preparative HPLC proved time-consuming due to the poor solubility of **1** and resulted in substantially lower yields of only 13% of **1** when performed on batches employing only double-digit milligram quantities of **28**. Collectively, the complementary nature of these methods—combining high product purity, improved solubility and functional handling, and enhanced stability and scalability—constitutes a substantial methodological improvement over existing synthetic approaches.

Thus, the selection of the preferred synthetic strategy ultimately depends on one’s own preferences and available equipment, as both routes constitute highly efficient and well-suited approaches to DFO*, each enabling its reliable preparation in good yield and high purity without noticeable fragmentation. This represents a significant advancement over the current literature, making the chelator more readily accessible and paving the way for its broader applicability in the development and production of ^89^Zr-labeled PET radiopharmaceuticals.

## 3. Materials and Methods

### 3.1. General: Chemicals, Solvents and Reagents

All solvents and reagents were purchased from commercial sources in at least analytical grade quality and used without further purification. *Tert*-butyl-(5-hydroxypentyl)carbamate and *O*-(tetrahydro-2*H*-pyran-2-yl)hydroxylamine were purchased from BLD Pharmatech (Kaiserslautern, Germany). Triphenylphosphine (PPh_3_), tetrabromomethane (CBr_4_), oxolane-2,5-dione, sodium carbonate, *N*,*N*-Diisopropylethylamine (DIPEA) and anhydrous dimethylformamide (DMF) were obtained from Merck (Darmstadt, Germany). Anhydrous acetonitrile (MeCN) and tetrahydrofuran (THF) were obtained from Thermo Fisher scientific (Schwerte, Germany). Deferrioxamine (DFO) mesylate was purchased from ABCR (Karlsruhe, Germany). *O*-(7-azabenzotriazol-1-yl)-*N*,*N*,*N*′,*N*′-tetramethyluronium hexafluoro-phosphate (HATU) was purchased from Genscript (Rho, Italy). Trifluoroacetic acid (TFA), petroleum ether (PE, 40–60 °C), potassium carbonate, triethylamine (NEt_3_), hydrochloric acid, anhydrous sodium sulfate (Na_2_SO_4_), acetone, and diethyl ether (Et_2_O) were obtained from Carl Roth (Karlsruhe, Germany). Dichloromethane (DCM), methanol (MeOH), and ethyl acetate (EtOAc) were obtained from Häberle Labortechnik (Lonsee-Ettlenschieß, Germany). Deionized water used for HPLC was produced by the water purifier Aquinity^2^ P10 from membraPure GmbH (Berlin, Deutschland). Instrumentation. Synthware round-bottom pressure vessels with PTFE bushing were obtained from Merck (Darmstadt, Germany). Analytical HPLC-ESI-MS and HPLC: for analytical HPLC-MS, a Waters ARC system with Acquity QDa mass detector was used, equipped with an XBridge C18 column (3.5 μm, 4.6 × 50 mm), Waters, Eschborn, Germany). For analytical HPLC chromatography, a Chromolith Performance (RP-18e, 100–4.6 mm, Merck, Darmstadt, Germany), for semipreparative analyses and purifications, a Chromolith (RP-18e, 100–10 mm, Merck, Darmstadt, Germany), and for preparative purifications, an XBridge Peptide BEH C18 (130 Å, 5 μm, 10 mm × 250 mm) column (Waters, Eschborn, Germany) were used together with a Dionex UltiMate 3000 system (Dionex, Dreieich, Germany), respectively. Analytical and semipreparative HPLC were performed with a flow rate of 4 mL/min and preparative HPLC with a flow rate of 6 mL/min. Reverse-phase flash column chromatography was done on an automated Puriflash XS530 system (Interchim, Montluçon, France) together with C18 columns of appropriate dimensions using H_2_O and MeCN as the eluents. Nuclear magnetic resonance (NMR) spectroscopy was carried out on a 500 MHz Varian NMR System or a 300 MHz MERCURYplus NMR spectrometer (Agilent Technologies, Waldbronn, Germany). MALDI-TOF-MS: Matrix-Assisted Laser Desorption/Ionization (MALDI) time-of-flight mass spectra were obtained utilizing a Bruker Daltonics Microflex spectrometer (Bruker, Bremen, Germany), linear acquisition mode, positive ion source. HR-ESI-MS: For high-resolution electrospray ionization mass spectroscopy a Bruker microTOF-Q II ESI spectrometer (Bremen, Germany) was used.

### 3.2. Stability Studies of DFO Mesylate, ***1*** and ***11***

DFO mesylate and *^t^*Bu-Boc-DFO* (**11**) (obtained as described before [15]) were each dissolved in water to afford 20 mM stock solutions whereas DFO* (**1**) was dissolved in DMSO to yield a stock solution of the same concentration. For stability measurements, 5 µL of each 20 mM stock solution were diluted with 95 µL of the respective test solution (0.1%, 1%, 10%, and 100% TFA; 0.1%, 1%, 10%, and 100% formic acid; water; 10 mM ammonium bicarbonate; 1 M and 32% hydrochloric acid) and incubated at ambient temperature. Aliquots of 10 µL of each reaction mixture were analyzed after 1 h, 3 h, and 24 h by analytical HPLC (conditions: column: Chromolith Performance RP-18e (100 × 4.6 mm, Merck); eluents: water and MeCN (90%) containing 10 mM NH_4_HCO_3_; gradient: 0.0–4.0 min, 0–45% MeCN). Peak integrals of the analytes were compared to those of freshly prepared reference solutions in water (5 µL of a 20 mM stock solution diluted with 95 µL water). All experiments were performed in triplicate using independently prepared reaction mixtures.

### 3.3. Chemical Syntheses

**16** (*tert*-butyl-(5-bromopentyl)carbamate). *Tert*-butyl-(5-hydroxypentyl)carbamate (2.5 g, 12 mmol) and triphenylphosphine (4.86 g, 18 mmol) were dissolved in THF. CBr_4_ (6.35 g, 19 mmol) was dissolved in THF and added dropwise under ice bath cooling. The mixture was stirred at 0 °C for 30 min, then warmed up to room temperature and stirred overnight. After completion of the reaction, the solvent was removed under reduced pressure. The mixture was purified by column chromatography on silica gel (PE: EtOAc = 10:1, R_f_ = 0.34) to isolate **16** (2.5 g, 78%) as a colorless oil.


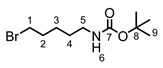


^1^H-NMR (500 MHz, CDCl_3_) *δ* = 4.52 (s, 1H, H6), 3.39 (t, ^3^*J* = 7 Hz, 2H, H1), 3.11(t, ^3^*J* = 6.5 Hz, 2H, H5), 1.89–1.84 (m, 2H, H2), 1.51–1.43 (m, 13H, H3, H4, and H9); ^13^C-NMR (125 MHz, CDCl_3_, APT) *δ* = 156.11 (C7), 79.28 (C8), 40.48 (C5), 33.73 (C1), 32.45 (C2), 29.41 (C4), 28.55 (C9), 25.47 (C3); HR-ESI-MS (*m*/*z*) for [M + Na]^+^ (calculated): 288.0572 (288.0570).

**17** (*tert*-butyl (5-(((tetrahydro-2*H*-pyran-2-yl)oxy)amino)pentyl)carbamate). **16** (1.02 g, 3.8 mmol) and K_2_CO_3_ (2.15 g, 15.3 mmol) were dissolved/suspended in MeCN (30 mL). *O*-(tetrahydro-2*H*-pyran-2-yl)hydroxylamine (0.89 g, 7.6 mmol) was added and the mixture was stirred at 60 °C overnight. After complete reaction, the solvent was removed under reduced pressure. The mixture was purified by column chromatography on silica gel (DCM:MeOH = 100:1, R_f_ = 0.16) to isolate **17** as a colorless oil in 40% yield (0.46 g, 1.41 mmol).


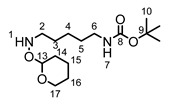


^1^H-NMR (500 MHz, CDCl_3_) *δ* = 4.92–4.90 (m, 1H, H13), 4.58 (s, 1H, H7), 3.92–3.88 (m, 1H, H17′), 3.59–3.55 (m, 1H, H17′), 3.11–3.07 (m, 2H, H6), 3.05–2.95 (m, 2H, H2), 1.78–1.66 (m, 2H, H14′ and H15′), 1.62–1.45 (m, 8H, H3, H5, H14′, H15′ and H16), 1.41 (s, 9H, H10), 1.38–1.32 (m, 2H, H4); ^13^C-NMR (125 MHz, CDCl_3_, APT) *δ* = 156.09 (C8), 101.33 (C13), 79.14 (C9), 63.14 (C17), 51.65 (C2), 40.50 (C6), 32.37 + 29.96 (C5), 28.98 (C14), 28.52 (C10), 26.44 (C3), 25.28 (C16), 24.39 + 23.03 (C4), 19.90 (C15). HR-ESI-MS (*m*/*z*) for [M + Na]^+^ (calculated): 325.2093 (325.2098).

**18** (4-((5-((*tert*-butoxycarbonyl)amino)pentyl)((tetrahydro-2*H*-pyran-2-yl)oxy)amino)-4-oxobutanoic acid). **17** (161.1 mg, 0.53 mmol), triethylamine (143 µL, 1.07 mmol) and succinic anhydride (80.0 mg, 0.80 mmol) were dissolved in MeCN (10 mL). The mixture was stirred at room temperature overnight. After complete reaction, the solvent was removed under reduced pressure. The residue was dissolved in Et_2_O and washed with saturated NaHCO_3_ solution (3 × 10 mL). The aqueous phase was collected and the pH was adjusted to 4 using 10% HCl solution (ca. 20 mL). The aqueous phase was subsequently extracted with DCM (3 × 10 mL), and the combined organic phases were dried with Na_2_SO_4_. The solvent was removed under reduced pressure to isolate **18** as a white solid in 92% yield (197.3 mg, 0.46 mmol).


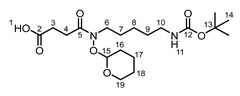


^1^H-NMR (500 MHz, CDCl_3_) *δ* = 5.96 (s, 1H, H11′), 4.88 (s, 1H, H15), 4.70 (s, 1H, H11′), 3.97–3.93 (m, 1H, H19′), 3.90–3.77 (m, 1H, H6′), 3.73–3.63 (m, 1H, H6′), 3.61–3.56 (m, 1H, H19′), 3.10–3.06 (m, 2H, H10), 2.90 (s, 1H, H4′), 2.71–2.61 (m, 3H, H3 and H4′), 1.83–1.76 (m, 2H, H16′ and H17′), 1.68–1.59 (m, 6H, H7, H18, H16′ and H17′), 1.51–1.38 (m, 11H, H9 and H14), 1.30–1.24 (m, 2H, H8); ^13^C-NMR (125 MHz, CDCl_3_, APT) *δ* = 177.2 (C2), 176.61 (C5), 156.22 (C12), 104.43 + 103.94 (C15), 80.52 + 79.33 (C13), 63.97 (C19), 45.64 + 45.95 (C6), 41.76 + 40.58 (C10), 32.44 + 29.60 (C9), 29.24 (C16), 29.04 (C3), 28.55 (C14), 27.87 + 27.63 (C4), 26.78 + 26.59 (C7), 25.02 (C18), 23.82 + 23.47 (C8), 19.85 (C17); HR-ESI-MS (*m*/*z*) for [M + Na]^+^ (calculated): 425.2252 (425.2258).

**19** (*tert*-butyl (3,14,25-trihydroxy-2,10,13,21,24,32,35-heptaoxo-36-((tetrahydro-2*H*-pyran-2-yl)oxy)-3,9,14,20,25,31,36-heptaazahentetracontan-41-yl)carbamate). **18** (200 mg, 0.5 mmol), HATU (180.1 mg, 0.5 mmol) and DIPEA (175 µL, 1.0 mmol) were dissolved in dried DMF (10 mL) and the mixture was stirred for 15 min at ambient temperature. Subsequently, DFO mesylate (215.6 mg, 0.33 mmol) was added and the mixture was stirred at 40 °C for 2 days. After completion of the reaction, cold acetone (10 mL) was added to induce precipitation of the product. The precipitate was collected by centrifugation (3500 rcf, 15 min, 4 °C) and washed with cold acetone (3 × 10 mL) to isolate **19** (226.1 mg, 68%) as a white solid. The crude product (purity of 90%) was used for next step without further purification.


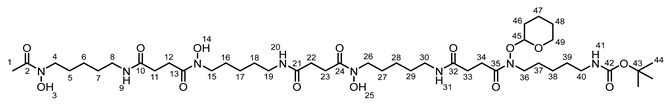


^1^H-NMR (500 MHz, DMSO-*d*_6_) *δ* = 9.65–9.60 (m, 3H, H3, H14 and H25), 7.77 (t, ^3^*J* = 5.5 Hz, 3H, H9, H20 and H31), 6.74 (t, ^3^*J* = 5.5 Hz, 1H, H41), 4.96 (s, 1H, H45), 3.89–3.85 (m, 1H, H49′), 3.72–3.66 (m, 1H, H36′), 3.58–3.53 (m, 1H, H49′), 3.49–3.44 (m, 7H, H4, H15, H26 and H36′), 3.02–2.98 (m, 6H, H8, H19 and H30), 2.88 (q, ^3^*J* = 6.5 Hz, 2H, H40), 2.77–2.73 (m, 1H, H34′), 2.64–2.53 (m, 5H, H12, H23 and H34′), 2.30–2.25 (m, 6H, H11, H22 and H33), 1.96 (s, 3H, H1), 1.73–1.72 (m, 2H, H46′ and H47′), 1.55–1.49 (m, 12H, H5, H16, H27, H37, H46′, H47′ and H48), 1.41–1.32 (m, 16H, H7, H18, H29, H39′, and H44), 1.22–1.16 (m, 9H, H6, H17, H28, H38, and H39′); ^13^C-NMR (125 MHz, DMSO-d_6_, APT) *δ* = 171.98 (C2), 171.33 (C10, C21, and C32), 171.03 (C13 and C24), 170.15 (C35), 155.59 (C42), 103.45 (C45), 77.31 (C43), 62.90 (C49), 47.10 (C36), 46.80 (C4, C15, and C26), 38.80 (C40), 38.43 (C8, C19, and C30), 29.93 (C33), 29.70 (C22 and C11), 29.14 (C39), 28.82 (C7, C18, and C29), 28.60 (C46), 28.27 (C44), 27.67 (C12 and C23), 27.58 (C34), 26.66 (C37), 26.04 (C5, C16, and C27), 24.57 (C48), 23.50 (C6, C17, and C28), 22.88 (C38), 20.35 (C1), 19.25 (C47); HR-ESI-MS (m/z) for [M + Na]^+^ (calculated): 967.5697 (967.5686).

**1** (DFO*) (prepared by synthesis pathway A)**. 19** (226.1 mg) was dissolved in a mixture of TFA and DCM (TFA:DCM = 20:80 (*v*:*v*), 2 mL) and reacted in an ultrasonic bath at 20 °C for 5 min. The volatile components were removed and the crude product was purified by preparative HPLC (0–50% MeCN + 0.1% TFA in 8 min, R_t_ = 6.4 min, 85.6 mg), giving the product in 55% yield and 98% purity. Care should be taken to avoid the product to stand uncooled in solution after purification.

**1** (prepared by synthesis pathway B). TBAF in THF (1.0 M, 30.0 mL, 30.0 mmol, 37 eq.) was added to **28** (1.00 g, 820 µmol) and the resulting solution was stirred for 20 min at ambient temperature. Methanol (20 mL) was added and the mixture poured on ice cold diethyl ether (300 mL). The resulting mixture was stored at 3 °C for 16 h, the precipitate centrifuged, collected and washed with diethyl ether (3 × 40 mL). After drying, the product was obtained in 98% yield and 80% purity. Afterwards, **1** was recrystallized from boiling 2-propanol (500 mL), collected by centrifugation and washed with 2-propanol (3 × 40 mL), followed by diethyl ether (40 mL). After drying, the product was obtained as a light orange solid in 72% yield (452 mg, 594 µmol) and 90% purity, which is sufficient for further reaction. **1** could be further purified by RP flash chromatography using H_2_O and MeCN as the eluents (column: PF-15C18HQ-F0080, gradient: 0.0–50.0 min 0.0–35% MeCN, R_t_ = 20 min). After lyophilization, **1** was obtained as a white solid in 34% yield (209.9 mg, 276 µmol) and 98% purity.


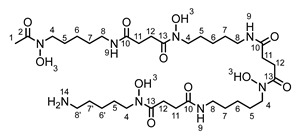


^1^H-NMR (300 MHz, DMSO-*d*_6_) *δ* = 9.66 (bs, 2H, H3), 7.78 (bm, 3H, H9 + H14), 3.45 (m, 8H, H4), 3.00 (q, ^3^*J* = 6.5 Hz, 6H, H8), 2.76 (q, ^3^*J* = 6.6 Hz, 2H, H8′), 2.57 (t, ^3^*J* = 7.6 Hz, 6H, H12), 2.27 (t, ^3^*J* = 7.6 Hz, 6H, H11), 1.96 (s, 3H, H1), 1.48 (m, 10H, H5 + H7′), 1.37 (p, ^3^*J* = 7.2 Hz, 6H, H7), 1.22 (p, ^3^*J* = 8.0 Hz, 8H, H6 + H6′); ^13^C-NMR (75 MHz, DMSO-*d*_6_) *δ* = 172.4 (C2), 171.8 (C10 + C13), 47.5 and 47.2 (C4), 39.2 (C8‘), 38.9 (C8), 30.3 and 29.2 (C11), 28.0 (C7), 27.1 (C12), 26.5 (C5), 23.9 (C6), 23.3 (C6‘), 20.8 (C1); HR-ESI-MS (*m*/*z*) for [M + H]^+^ (calculated): 761.4756 (761.4767).

**20** (5-((((9*H*-fluoren-9-yl)methoxy)carbonyl)amino)pentyl methanesulfonate). To an ice-cooled solution of (9*H*-fluoren-9-yl)methyl (5-hydroxypentyl)carbamate (1.02 g, 3.13 mmol) in DCM (100 mL) were added DIPEA (1.10 mL, 6.48 mmol, 2.1 eq.) and mesyl chloride (285 µL, 3.69 mmol, 1.2 eq.), and the resulting mixture was stirred for 3 h at 0 °C. The organic layer was washed with a solution of citric acid (0.2 M, 3 × 20 mL), dried over Na_2_SO_4_ and the volatile components evaporated under reduced pressure to give the product as a light yellow solid in 87% yield (1.10 g, 2.73 mmol).


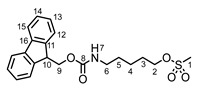


^1^H-NMR (300 MHz, DMSO-*d*_6_) *δ* = 7.88 (dt, ^3^*J* = 7.6 Hz, ^4^*J* = 0.9 Hz, 2H, H15), 7.69 (d, ^3^*J* = 7.4 Hz, 2H, H12), 7.41 (td, ^3^*J* = 7.7 Hz, ^4^*J* = 1.7 Hz, 2H, H14), 7.33 (td, ^3^*J* = 7.4 Hz, ^4^*J* = 1.3 Hz, 2H, H13), 7.29 (t, ^3^*J* = 5.8 Hz, 1H, H7), 4.32 (d, ^3^*J* = 6.8 Hz, 2H, H9), 4.20 (m, 3H, H10 + H2), 3.16 (s, 3H, H1), 3.00 (q, ^3^*J* = 6.4 Hz, 2H, H6), 1.66 (p, ^3^*J* = 6.7 Hz, 2H, H3), 1.45 (m, 2H, H5), 1.34 (m, 2H, H4); ^13^C-NMR (75 MHz, DMSO-*d*_6_) *δ* = 156.1 (C8), 144.0 (C11), 140.8 (C16), 127.6 (C14), 127.0 (C13), 125.1 (C12), 120.1 (C15), 70.4 (C2), 65.2 (C9), 46.8 (C10), 40.0 (C6), 36.5 (C1), 28.8 (C5), 28.2 (C3), 22.2 (C4); HR-ESI-MS (*m*/*z*) for [M + Na]^+^ (calculated): 426.1354 (426.1346).

**22** (4-(((*tert*-butyldiphenylsilyl)oxy)amino)-4-oxobutanoic acid). *O*-(tert-butyldiphenylsilyl)hydroxylamine (513 mg, 1.89 mmol) and succinic anhydride (559 mg, 5.59 mmol, 3.0 eq.) were suspended in DCM (50 mL) and stirred for 2.5 h at ambient temperature. The volatile components of the mixture were evaporated under reduced pressure in the presence of celite (4.0 g) and the product was purified by flash chromatography using EtOAc and cyclohexane as the eluents (gradient: 0–4 min 0% EtOAc, 4–40 min 0–100% EtOAc, R_t_ = 21 min), giving **22** in form of a white solid in 90% yield (632 mg, 1.70 mmol).


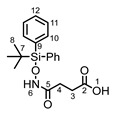


^1^H-NMR (500 MHz, DMSO-*d*_6_) *δ* = 12.12 (s, 1H, H1), 10.68 (s, 1H, H6), 7.69 (d, ^3^*J* = 5.1 Hz, 4H, H10), 7.46 (m, 2H, H12), 7.40 (m, 4H, H11), 2.29 (t, ^3^*J* = 7.1 Hz, 2H, H4), 2.13 (t, ^3^*J* = 7.1 Hz, 2H, H3), 1.05 (s, 9H, H8); ^13^C-NMR (125 MHz, DMSO-*d*_6_) *δ* = 173.5 (C2), 169.6 (C5), 135.4 (C10), 132.0 (C9), 130.0 (C12), 127.6 (C11), 28.8 (C4), 27.2 (C3), 26.7 (C8), 19.0 (C7); HR-ESI-MS (*m*/*z*) for [M–H]^−^ (calculated): 370.1475 (370.1480).

**23** (4-((5-((((9*H*-fluoren-9-yl)methoxy)carbonyl)amino)pentyl)((*tert*-butyldiphenylsilyl) oxy)amino)-4-oxobutanoic acid). To a solution of **27** (1.04 g, 1.80 mmol) in DCM (30 mL) was added succinic anhydride (578 mg, 5.78 mmol, 3.2 eq.) and the mixture was reacted for 16 h at ambient temperature. Diethyl ether (50 mL) was added and the solution was extracted with saturated NaHCO_3_ solution (3 × 25 mL). The aqueous solution was acidified to pH 1 using concentrated HCl (10 mL) and extracted with diethyl ether (3 × 25 mL). The combined organic phases were dried over sodium sulfate, and the volatile components evaporated under reduced pressure, giving the product as a colorless oil in 83% yield (1.01 g, 1.49 mmol). Care has to be taken to store the product at −20 °C to avoid decomposition.


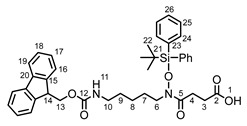


^1^H-NMR (500 MHz, DMSO-*d*_6_) *δ* = 10.69 (br s, 1H, H1), 7.88 (d, ^3^*J* = 7.6 Hz, 2H, H19), 7.67 (m, 3H, H16 and H24), 7.68 (d, ^3^*J* = 7.1 Hz, 2H, H20), 7.61 (d, ^3^*J* = 6.7 Hz, 4H, H28), 7.47 (m, 2H, H30), 7.40 (m, 6H, H22 and H28), 7.37 (m, 11H, H11, H17, H18, H25, and H26), 4.30 (d, ^3^*J* = 6.9 Hz, 2H, H13), 4.21 (t, ^3^*J* = 7.1 Hz, 1H, H14), 3.49 (t, ^3^*J* = 7.3 Hz, 2H, H6), 2.95 (m, 2H, H10), 2.58 (t, ^3^*J* = 6.8 Hz, 2H, H3), 2.40 (t, ^3^*J* = 6.7 Hz, 2H, H4), 1.51 (m, 2H, H7), 1.40 (q, ^3^*J* = 6.9 Hz, 2H, H7), 1.23 (m, 2H, H8), 0.99 (s, 9H, H22); ^13^C-NMR (125 MHz, DMSO-*d*_6_) *δ* = 174.1 (C2), 171.95 (C5), 156.06 (C12), 143.94 (C15), 140.73 (C20), 134.7 (C24), 130.0 (C26), 127.7 (C18), 127.62 (C23/C25), 127.56 (C23/C25), 127.0 (C17), 125.1 (C16), 120.1 (C19), 65.2 (C13), 46.8 (C13), 40.1 (C14), 29.0 (C9 and C4), 27.2 (C3), 26.4 (C22), 26.0 (C7), 23.3 (C8), 18.5 (C21); HR-ESI-MS (*m*/*z*) for [M + H]^+^ (calculated): 678.3205 (678.3125).

**25** ((9*H*-fluoren-9-yl)methyl 3,4-dihydropyridine-1(2*H*)-carboxylate). (9*H*-Fluoren-9-yl)methyl-(5-hydroxypentyl)carbamate (11.6 g, 35.7 mmol) was suspended together with neutral aluminum oxide (3.65 g) in DCM (200 mL). To this mixture, pyridinium chlorochromate (11.6 g, 54.0 mmol, 1.5 eq.) was added in one portion. The initially yellow to orange suspension turned black within a few minutes and was further reacted for 16 h at ambient temperature. The suspension was filtered over silica gel (5 cm), which was washed afterwards with several portions of DCM (overall 1 L). The volatile components of the light green solution were removed under reduced pressure, and the product was obtained as a light green solid in 82% yield (8.92 g, 29.2 mmol).


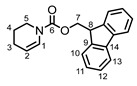


^1^H-NMR (500 MHz, CDCl_3_) *δ* = 7.78 (d, ^3^*J* = 7.5 Hz, 2H, H13), 7.60 (d, ^3^*J* = 7.4 Hz, 2H, H10), 7.41 (t, ^3^*J* = 7.4 Hz, 2H, H12), 7.33 (t, ^3^*J* = 7.4 Hz, ^4^*J* = 1.3 Hz, 2H, H11), 6.86 (dd, ^3^*J* = 19.2 Hz, ^3^*J* = 8.4 Hz, 1H, H1), 4.96 (m, 1H, H2), 4.44 (m, 2H, H7), 4.28 (t, ^3^*J* = 7.2 Hz, 1H, H8), 3.65 (m, 2H, H5), 2.08 (m, 2H, H3), 1.86 (m, 2H, H4); ^13^C-NMR (125 MHz, CDCl_3_) *δ* = 153.3 (C6), 144.0 (C9), 141.5 (C14), 127.9 (C12), 127.2 (C11), 125.2 (C10), 124.9 (C1), 120.2 (C13), 107.0 (C2), 68.0 (C7), 47.3 (C8), 42.4 (C5), 21.8 (C3), 21.6 (C4); HR-ESI-MS (*m*/*z*) for [M + Na]^+^ (calculated): 328.1315 (328.1308).

**26** ((9*H*-fluoren-9-yl)methyl-(5-(((*tert*-butyldiphenylsilyl)oxy)imino)pentyl)carbamate). **25** (7.39 g, 24.2 mmol), *O*-(*tert*-butyldiphenylsilyl)hydroxylamine (8.33 g, 30.7 mmol, 1.3 eq.), magnesium sulfate (11.5 g, 95.2 mmol, 4.2 eq.), and pyridinium *p*-toluenesulfonate (207 mg, 0.810 mmol, 3.6 mol%) were suspended in toluene (110 mL) and the reaction mixture was heated to 110 °C in a sealed pressure vessel for 20 h. After cooling to ambient temperature, the solid was filtered off and the solvent removed under reduced pressure. The product was purified by column chromatography (eluent: 10% EtOAc in cyclohexane, R_f_ = 0.15) and obtained in 93% yield as a mixture of *E*/*Z* isomers (0.61/0.39) (13.0 g, 33.6 mmol).


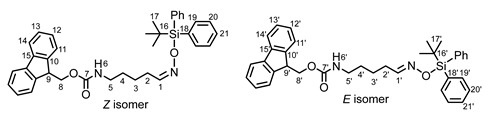


^1^H-NMR (500 MHz, DMSO-*d*_6_) *δ* = 7.88 (d, ^3^*J* = 7.6 Hz, 2H, H14 + H14′), 7.77 (t, ^3^*J* = 6.1 Hz, 0.6H, H1, *E* isomer), 7.68 (d, ^3^*J* = 7.1 Hz, 2H, H11 + H11′), 7.64 (m, 4H, H19 + H19′), 7.40 (m, 8H, H13 + H13′, H20 + H20′, H21 + H21′), 7.31 (t, ^3^*J* = 7.4 Hz, 2H, H12 + H12′), 7.26 (t, ^3^*J* = 5.8 Hz, 1H, H6 + H6′), 7.10 (t, ^3^*J* = 5.4 Hz, 0.4H, H1′, *Z* isomer), 4.31 (dd, ^3^*J* = 6.9 Hz, ^3^*J* = 4.8 Hz, 2H, H8 + H8′), 4.20 (t, ^3^*J* = 6.9 Hz, 1H, H9 + H9′), 3.04 (q, ^3^*J* = 6.0 Hz, 0.8H, H5′, *Z* isomer), 2.96 (q, ^3^*J* = 6.1 Hz, 1.2H, H5, *E* isomer), 2.51 (q, ^3^*J* = 6.0 Hz, 0.8H, H2′, *Z* isomer), 2.16 (q, ^3^*J* = 6.5 Hz, 1.2H, H2, *E* isomer), 1.50 (m, 1.6H, H3′ + H4′, *Z* isomer), 1.38 (m, 2.4H, H3 + H4, *E* isomer), 1.02 (s, 9H, H17 + H17′); ^13^C-NMR (125 MHz, DMSO-*d*_6_) *δ* = 157.34, 157.32, 157.28 + 157.27 (C1 + C1′), 156.10 + 156.07 (C7 + C7′), 143.91 (C10 + C10′), 140.73 + 139.41 (C15 + C15′), 134.99 + 134.92 (C19 + C19′), 133.24 + 133.20 (C18 + C18′), 129.73 + 129.70 (C21 + C21′), 127.69, 127.66, 127.65, + 127.54 (C13 + C13′, C4 + C4′), 127.25 + 126.98 (C12 + C12′), 125.09 (C11 + C11′), 121.34 (C14 + C14′), 65.13 (C8 + C8′), 46.79 (C9 + C9′), 39.8 (C5′, *Z* isomer), 39.7 (C5, *E* isomer), 29.5 + 29.2 (C3, *E* isomer), 29.0 + 28.7 (C3′, *Z* isomer), 28.57 + 28.52 (C2, *E* isomer), 26.84 (C17, *E* isomer), 26.81 (C17′, *Z* isomer), 24.84 + 24.8 (C2′, *Z* isomer), 23.1 + 23.0 (C4, *E* isomer), 22.8 + 22.7 (C4′, *Z* isomer), 18.85 + 18.80 (C16 + C16′); HR-ESI-MS (*m*/*z*) for [M + Na]^+^ (calculated): 599.2697 (599.2706).

**27** ((9*H*-fluoren-9-yl)methyl (5-(((*tert*-butyldiphenylsilyl)oxy)amino)pentyl)carbamate). **26** (10.6 g, 18.3 mmol) was dissolved in THF (120 mL) in a pressure vessel. To this solution, sodium cyanoborohydride (11.9 g, 189 mmol, 10 eq.) was first added, followed by acetic acid (2.67 g, 46.8 mmol, 2.5 eq.) before the vessel was sealed and warmed to 50 °C for 18 h. After cooling to ambient temperature, DCM (100 mL) was added and the combined organic phases were washed with saturated NaHCO_3_ solution (3 × 50 mL). The aqueous phase was extracted with DCM (3 × 100 mL), the combined organic phases were dried over sodium sulfate and the volatile components were evaporated under reduced pressure. The product was purified by column chromatography (eluent: 10% EtOAc in cyclohexane, R_f_ = 0.10) and obtained as a colorless crystalline solid in 56% yield (6.11 g, 10.6 mmol).


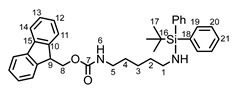


^1^H-NMR (300 MHz, CDCl_3_) *δ* = 7.76 (m, 6H, H20 + H14), 7.62 (d, ^3^*J* = 7.4 Hz, 2H, H11), 7.41 (m, 8H, H13 + H19 + H21), 7.34 (tt, ^3^*J* = 7.4 Hz, ^4^*J* = 1.5 Hz, 2H, H12), 4.72 (s, 1H, H6), 4.44 (d, ^3^*J* = 6.7 Hz, 2H, H8), 4.24 (t, ^3^*J* = 6.9 Hz, 1H, H9), 3.11 (q, ^3^*J* = 6.8 Hz, 2H, H5), 2.90 (t, ^3^*J* = 6.7 Hz, 2H, H1), 1.41 (m, 4H, H2 + H4), 1.23 (q, ^3^*J* = 7.1 Hz, 2H, H3), 0.99 (s, 9H, H17); ^13^C-NMR (75 MHz, CDCl_3_) *δ* = 156.5 (C7), 144.1 (C10), 141.4 (C15), 135.9 (C20), 134.1 (C18) 129.6 (C21), 127.8 (C13), 127.6 (C19), 127.1 (C12), 125.1 (C11), 120.1 (C14), 66.5 (C8), 54.0 (C1), 47.4 (C9), 41.0 (C5), 29.8 (C4), 27.5 (C17), 26.6 (C2), 24.4 (C3), 19.3 (C16); HR-ESI-MS (*m*/*z*) for [M + H]^+^ (calculated): 579.3060 (579.3037).

**28** ((9*H*-fluoren-9-yl)methyl (36-((*tert*-butyldiphenylsilyl)oxy)-3,14,25-trihydroxy-2,10,13,21,24,32,35-heptaoxo-3,9,14,20,25,31,36-heptaazahentetracontan-41-yl)carbamate). To a solution of **23** (1.03 g, 1.51 mmol, 1.2 eq.) and PyBOP (916 mg, 1.76 mmol, 1.4 eq.) in DMF (50 mL), DIPEA (417 µL, 2.45 mmol, 2.0 eq.) was added, and the mixture reacted for 60 min at ambient temperature. DFO mesylate (812 mg, 1.24 mmol, 1.0 eq.) was added and reacted for 90 min before the mixture was added to 3 °C cold diethyl ether (300 mL), forming a white precipitate. After 16 h at 3 °C, the solid was filtered and washed with 0.1% formic acid (3 × 60 mL) and diethyl ether (3 × 60 mL). The product was obtained as a white solid in 51% yield (770 mg, 0.631 mmol).


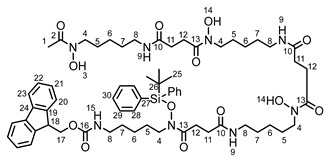


^1^H-NMR (500 MHz, DMSO-*d*_6_) *δ* = 9.64 (s, 1H, H3), 9.59 (s, 2H, H14), 7.88 (d, ^3^*J* = 7.6 Hz, 2H, H23), 7.77 (t, ^3^*J* = 6.4 Hz, 3H, H9), 7.68 (d, ^3^*J* = 7.1 Hz, 2H, H20), 7.61 (d, ^3^*J* = 6.7 Hz, 4H, H28), 7.47 (m, 2H, H30), 7.40 (m, 6H, H22 + H28), 7.32 (t, ^3^*J* = 7.4 Hz, 2H, H21), 7.23 (t, ^3^*J* = 5.8 Hz, 1H, H15), 4.29 (d, ^3^*J* = 7.0 Hz, 2H, H17), 4.20 (t, ^3^*J* = 7.0 Hz, 1H, H18), 3.45 (t, ^3^*J* = 6.7 Hz, 8H, H4), 2.99 (m, 8H, H8), 2.57 (t, ^3^*J* = 7.5 Hz, 6H, H12), 2.27 (t, ^3^*J* = 7.5 Hz, 6H, H11), 1.96 (s, 3H, H1), 1.49 (m, 8H, H5), 1.37 (q, ^3^*J* = 7.3 Hz, 8H, H7), 1.21 (m, 8H, H6), 1.09 (s, 9H, H25); ^13^C-NMR (125 MHz, DMSO-*d*_6_) *δ* = 172.0 (C13), 171.3 (C10), 170.7 (C2), 156.0 (C16), 143.9 (C19), 140.7 (C24), 135.5 (C28), 130.2 (C30), 127.8 (C22), 127.6 (C27/C29), 127.5 (C27/C29), 127.0 (C21), 125.1 (C20), 120.2 (C23), 65.2 (C17), 47.1 (C4), 46.8 (C18), 38.4 (C8), 29.9 (C11), 28.8 (C7), 27.6 (C12), 26.8 (C25), 26.0 (C5), 23.5 (C6), 20.3 (C1), 19.0 (C26); HR-ESI-MS (*m*/*z*) for [M + H]^+^ (calculated): 1221.6588 (1221.6626).

## 4. Conclusions

This work systematically demonstrates the pronounced acid lability of DFO and its derivative DFO*, thereby providing important insights into the successful synthesis, purification, and further conversion of these important chelating agents. Further, we present two novel synthetic routes to DFO*, the key precursor for biomolecule-conjugatable DFO* derivatives, both delivering the target compound in limited synthetic effort, good yield and exceptional purity, with no detectable decomposition of intermediates or product. Overall, this study advances the broader application of DFO* as the currently most suitable chelator for stably ^89^Zr-labeled biomolecules with favorable pharmacokinetic properties.

## Data Availability

The original contributions presented in the study are included in the article/Supplementary Materials; further inquiries can be directed to the corresponding author.

## Supplementary Materials

The following supporting information can be downloaded at: https://www.mdpi.com/article/10.3390/ph19060813/s1, containing analytical data of **1**, **16**–**20**, **22**, **23**, **25**–**28** (^1^H-, ^13^C-NMR and HR mass spectrometry data) (Figures S1–S24) and analytical HPLC chromatogram of the deprotection reaction of **11** to **1** (Figure S25).
