# Supplementary material for: New Synthetic Strategies Toward DFO*: Enhanced Yield and Purity of a Key Chelator for 89Zr Chemistry
Source: Pharmaceuticals (Basel). 2026 May 22;19(6):813. doi: 10.3390/ph19060813 (PMC13304809; doi:10.3390/ph19060813)
Supplement: Supplementary file 1 [file pharmaceuticals-19-00813-s001.zip › pharmaceuticals-4323158-supplementary.pdf]

Supplementary Information for manuscript

New Synthetic Approaches towards DFO*: Improving Yield and Purity of a Key Chelator for ^{89}Zr Chemistry

Nils F. Baier, Minqian Miao, Ralf Schirrmacher, Björn Wängler, Gert Fricker, Carmen Wängler

Content

Analytical data of **1**, **16 – 20**, **22**, **23**, **25 – 28** (^{1}H-, ^{13}C-NMR and HR mass spectrometry data) (Fig. S1 – S24)	page S2
Analytical HPLC chromatogram of the deprotection reaction of **11** to **1** (Fig. S25)	page S26

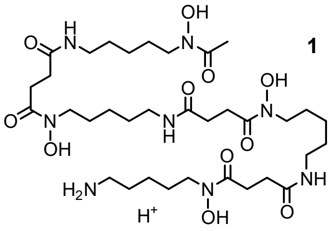

1

Chemical Formula: $C_{34}H_{65}N_8O_{11}^+$
Exact Mass: 761,4767

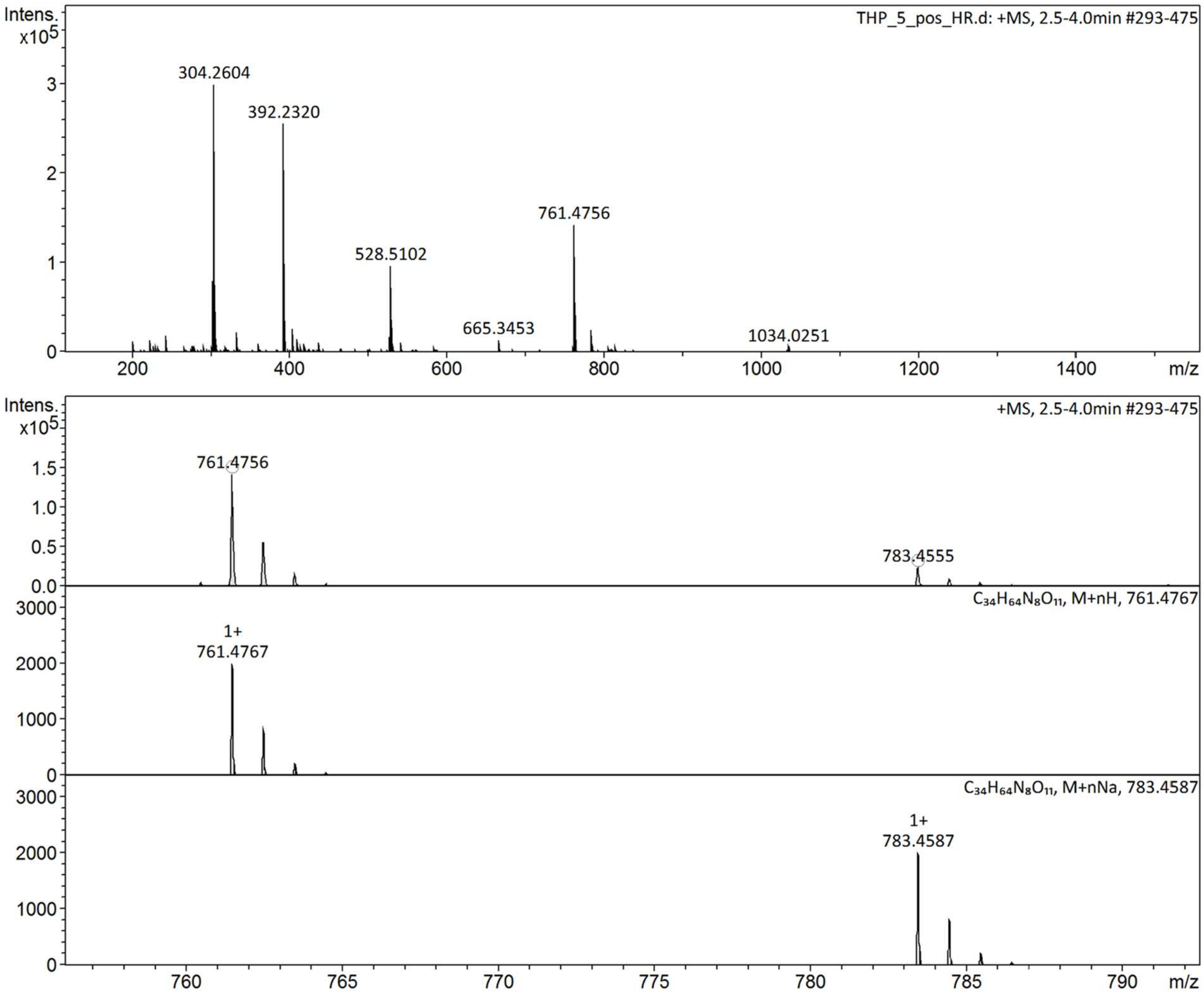

Figure S1: HR-ESI mass spectrum of **1**

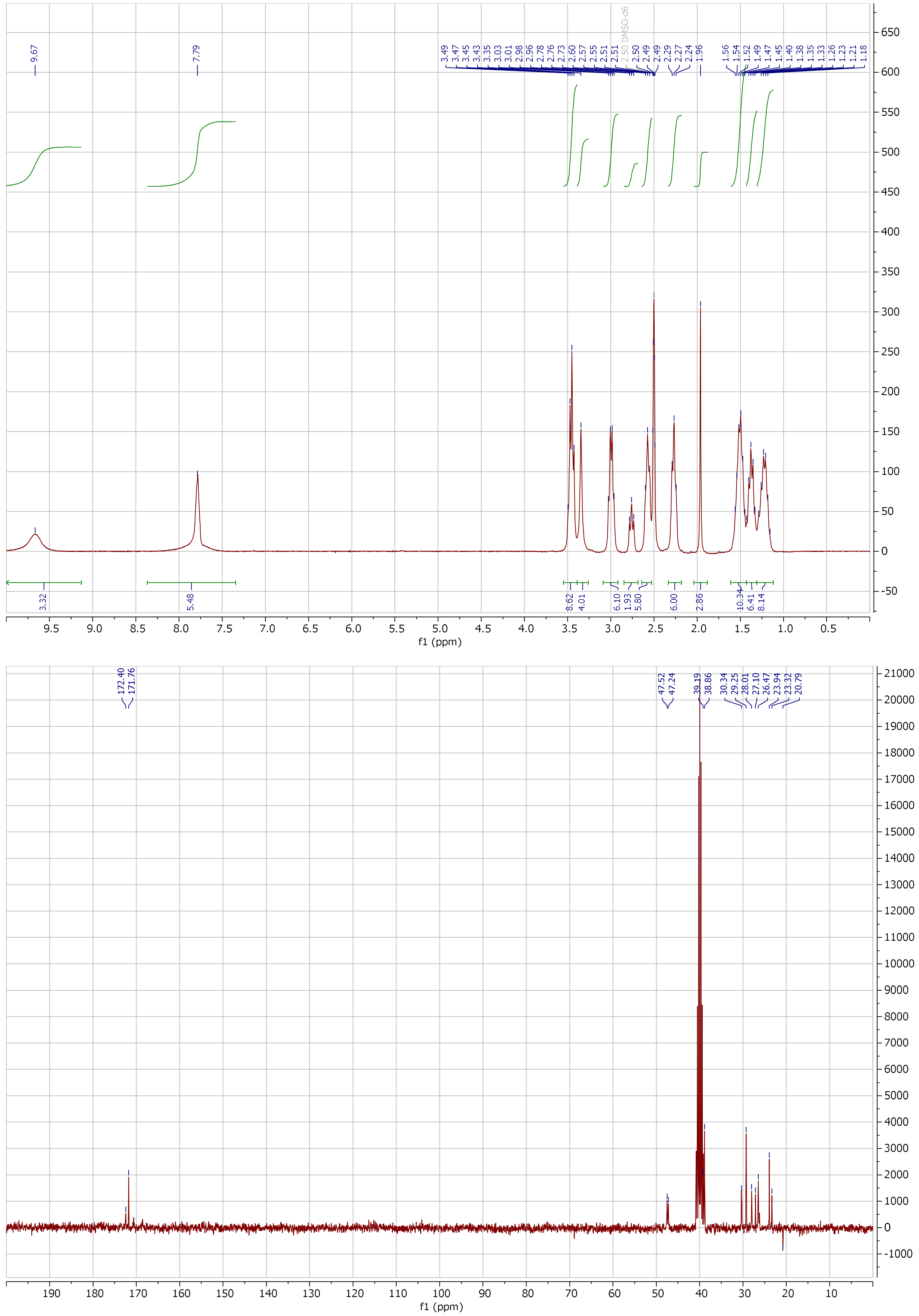

Figure S2: ^{1}H-NMR and ^{13}C-NMR of **1**

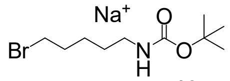

16

Chemical Formula: $C_{10}H_{20}BrNNaO_2^+$
Exact Mass: 288,0570

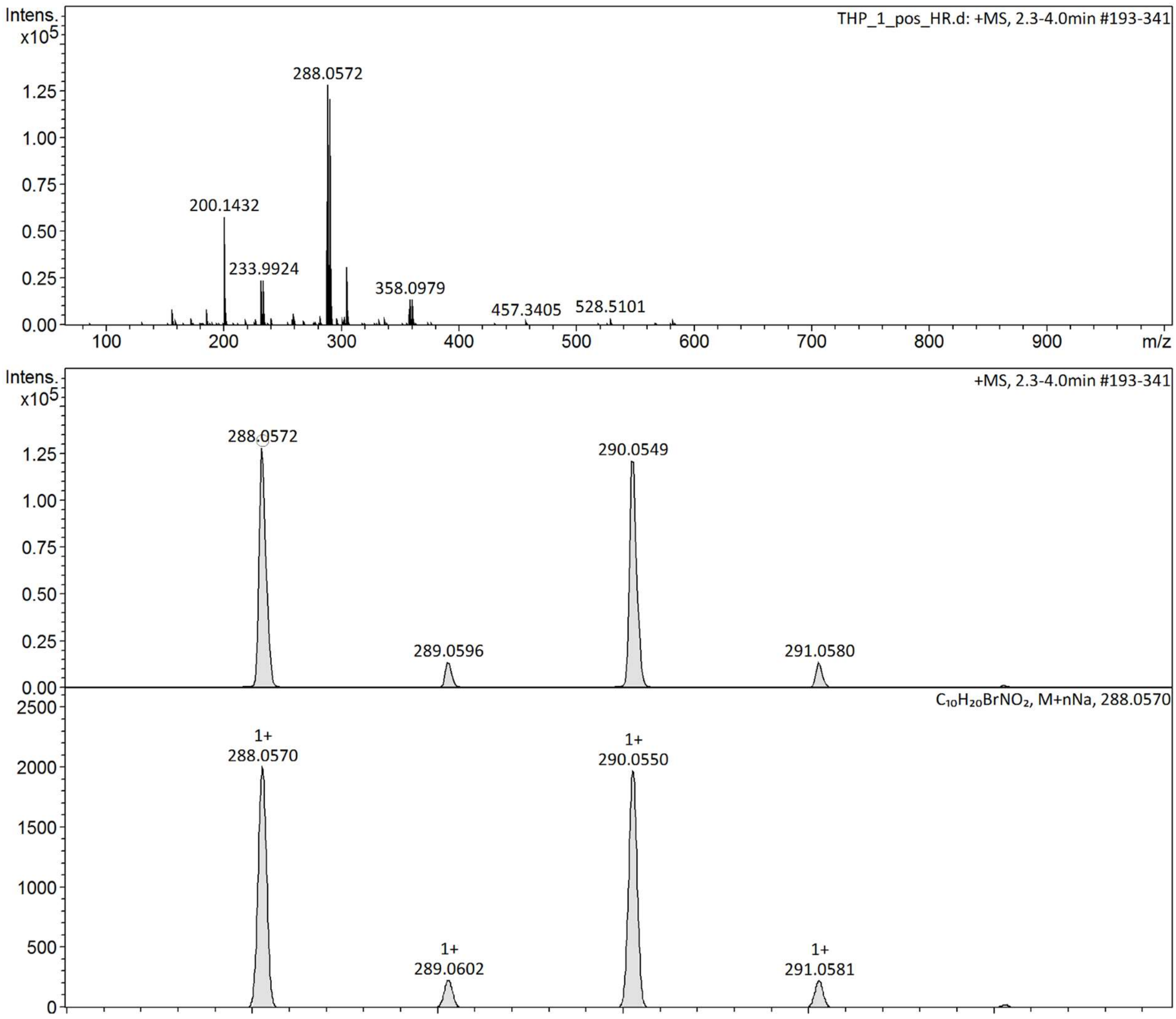

Figure S3: HR-ESI mass spectrum of **16**

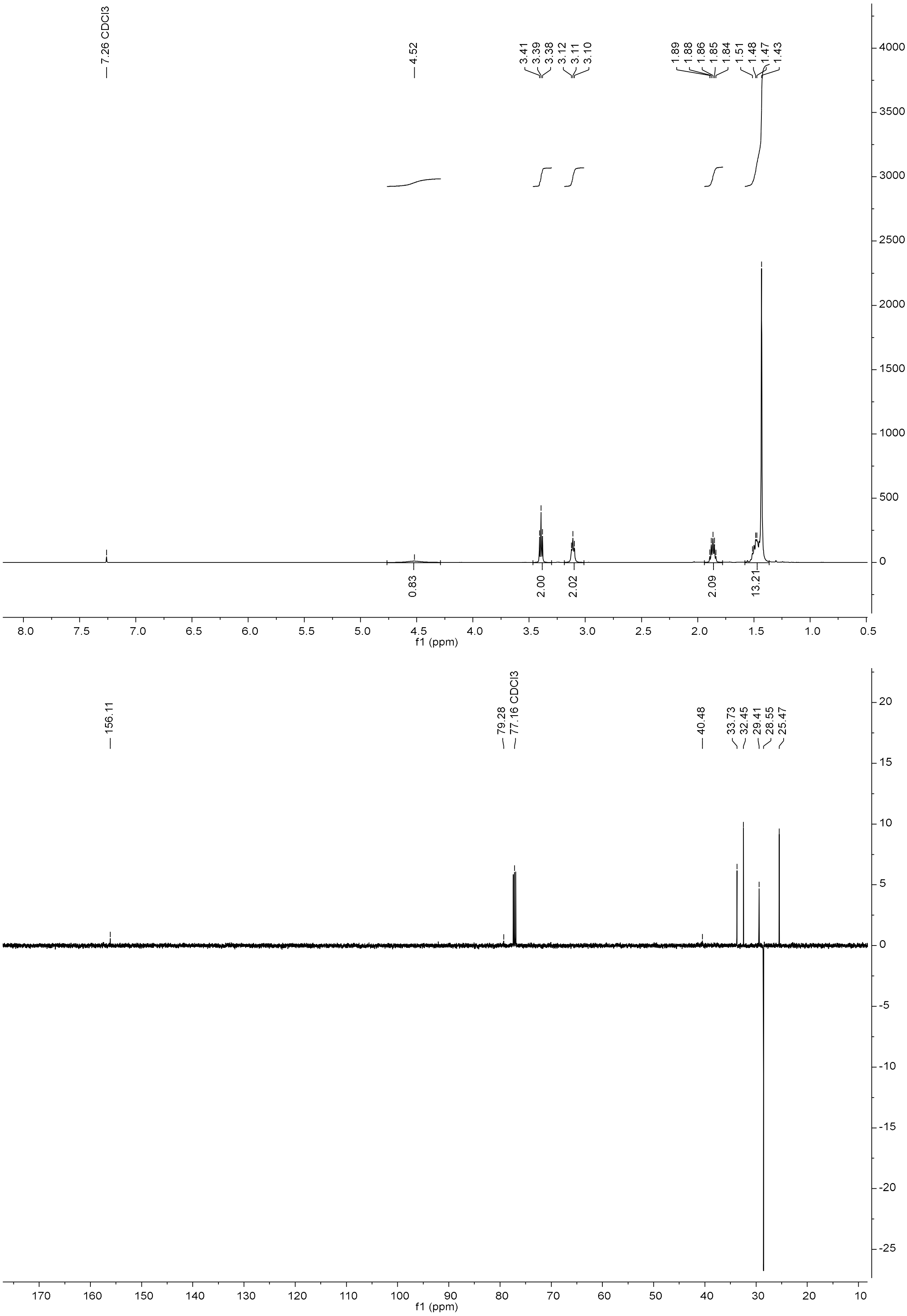

Figure S4: ^{1}H-NMR and ^{13}C-NMR of **16**

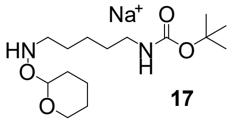

17

Chemical Formula: $C_{15}H_{30}N_2NaO_4^+$
Exact Mass: 325,2098

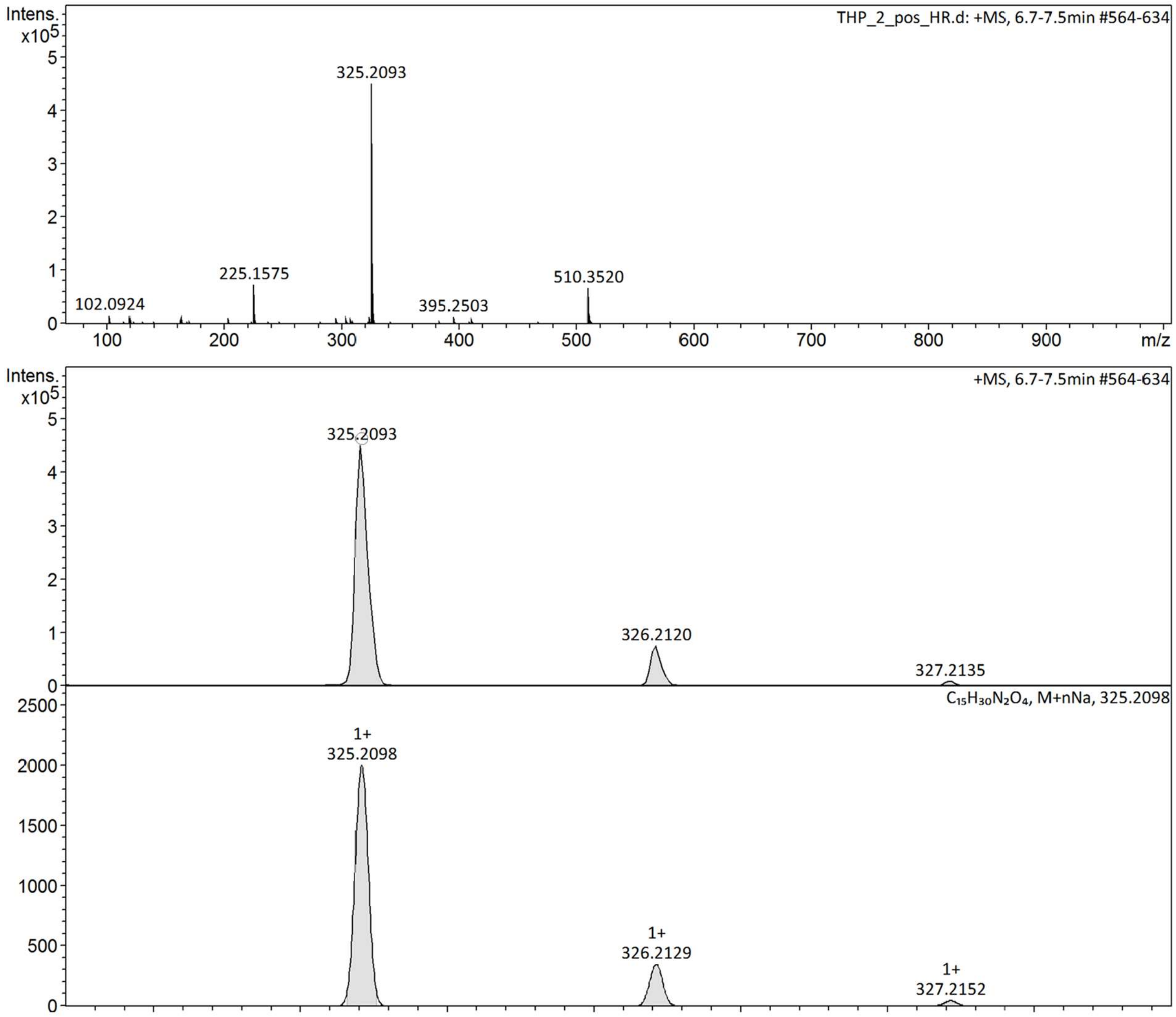

Figure S5: HR-ESI mass spectrum of **17**

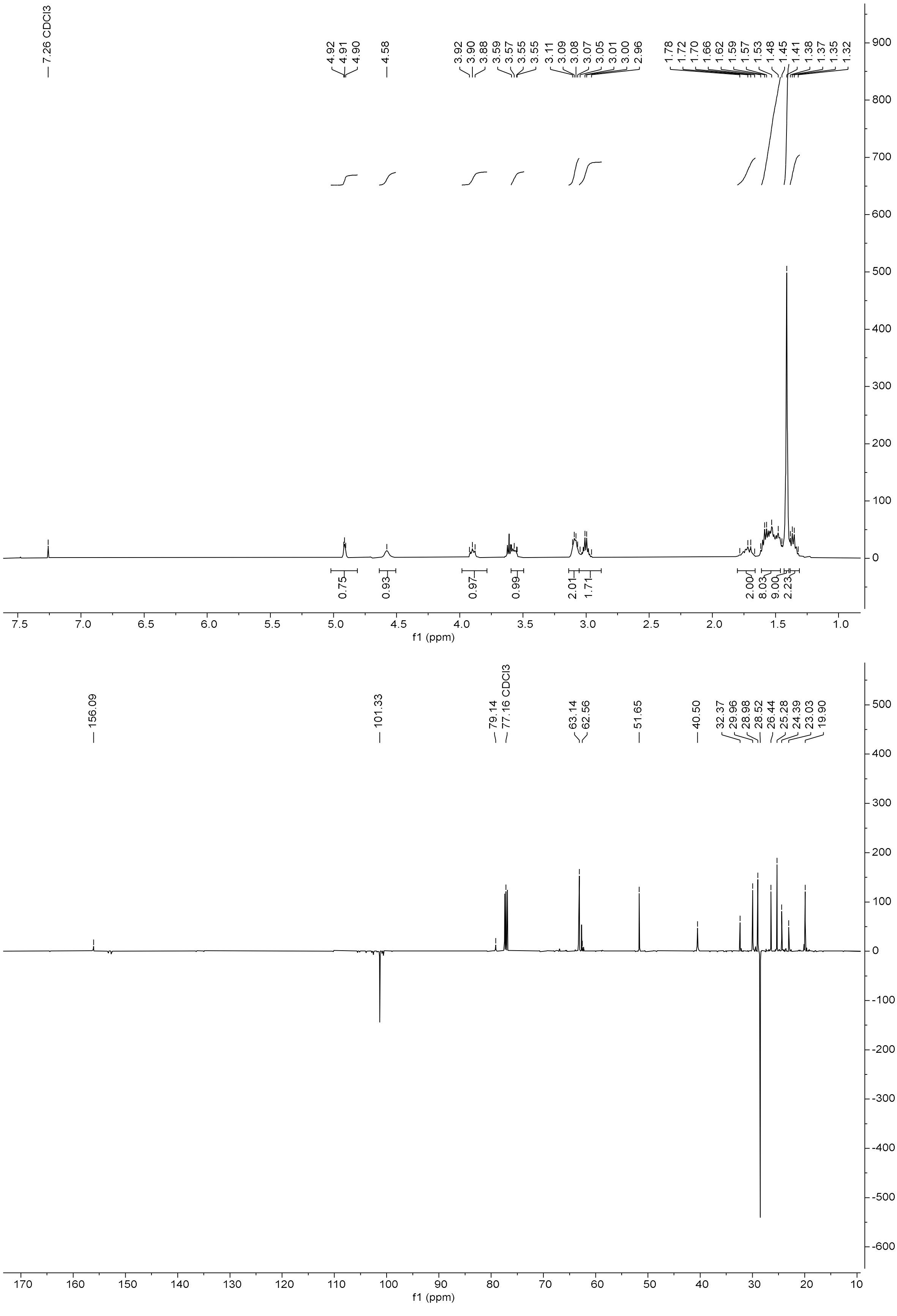

Figure S6: ^{1}H-NMR and ^{13}C-NMR of **17**

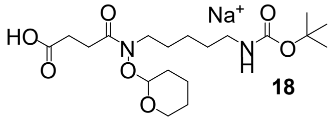

18

Chemical Formula: $C_{19}H_{34}N_2NaO_7^+$
Exact Mass: 425,2258

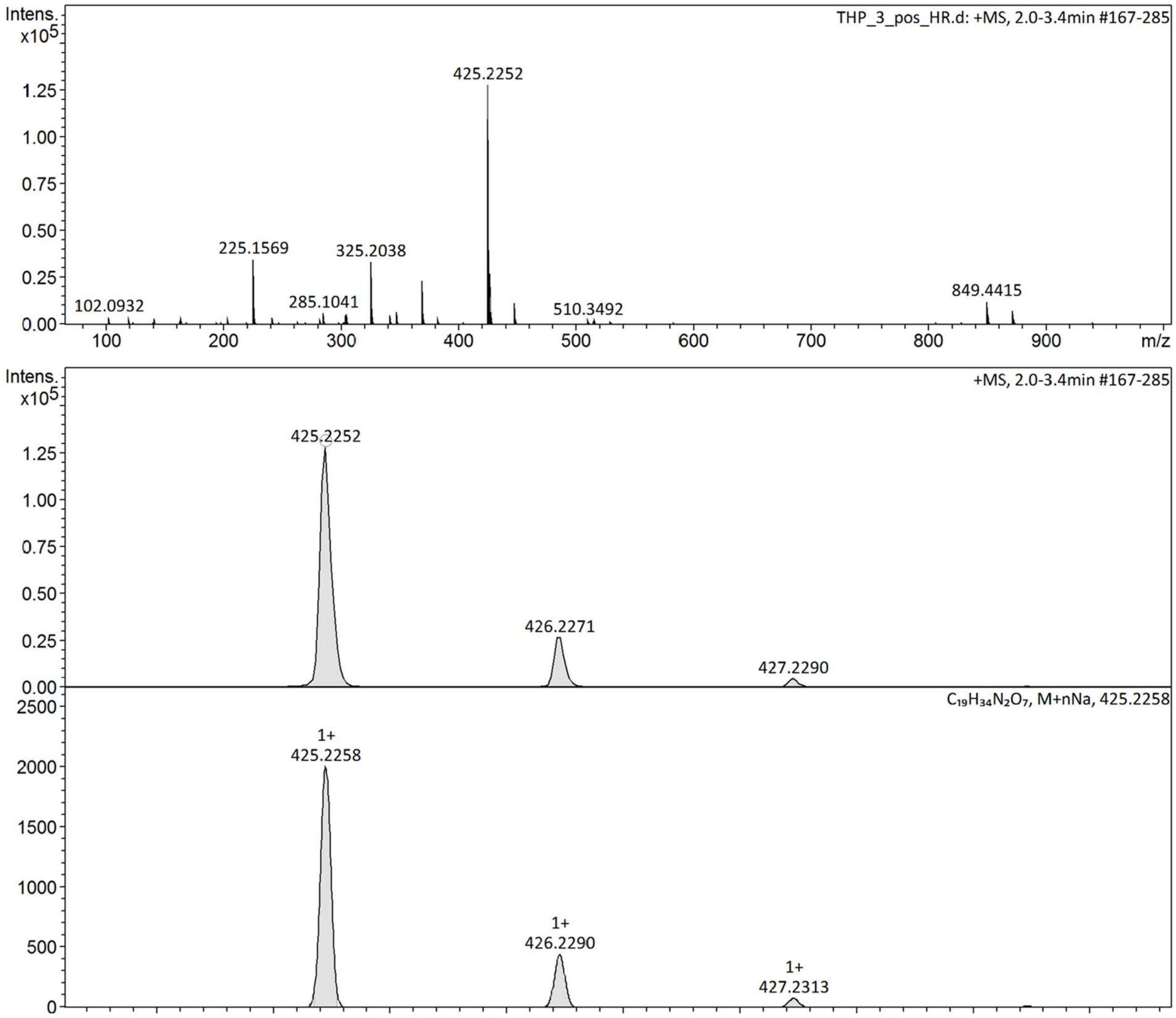

Figure S7: HR-ESI mass spectrum of **18**

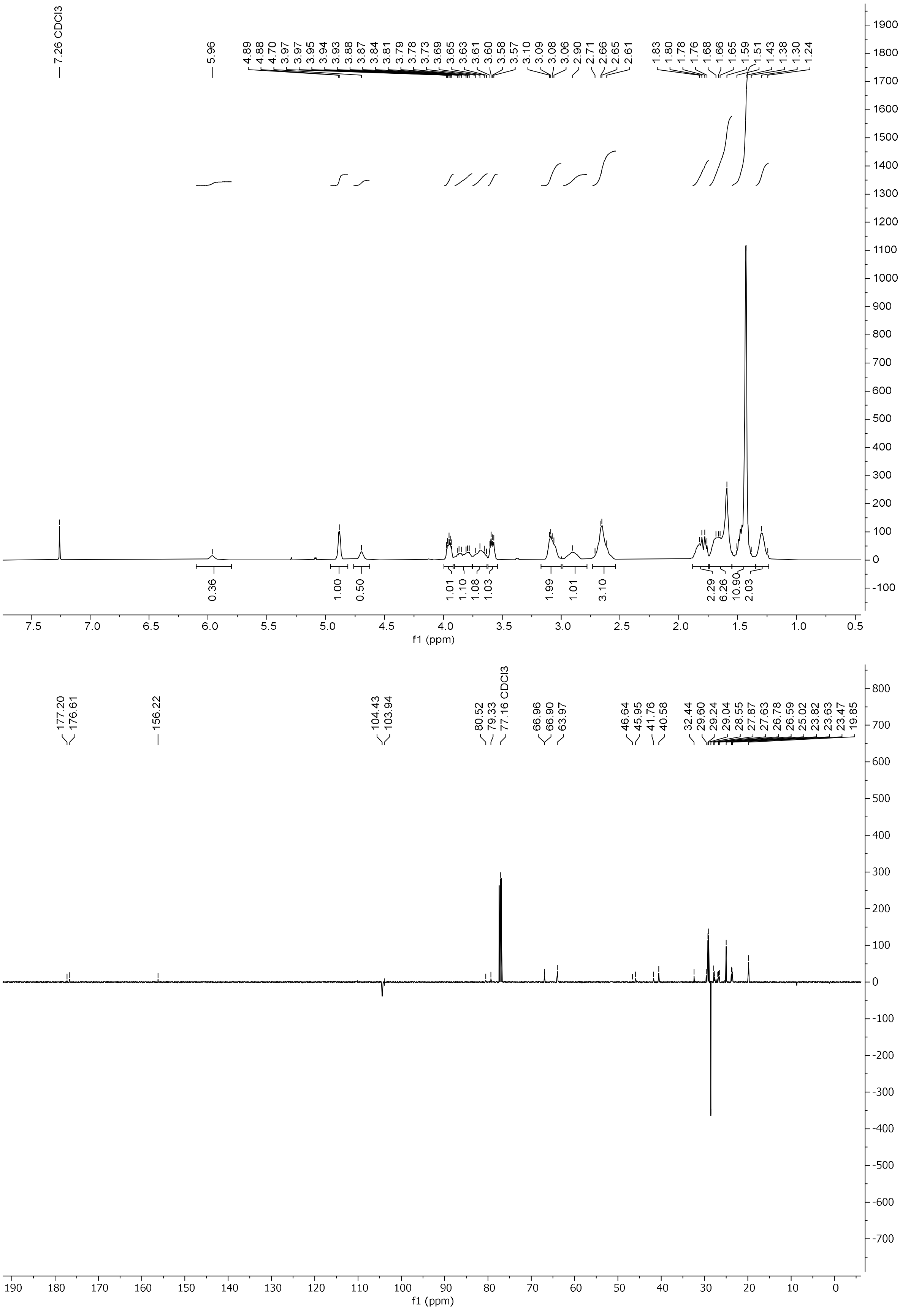

Figure S8: ^{1}H-NMR and ^{13}C-NMR of **18**

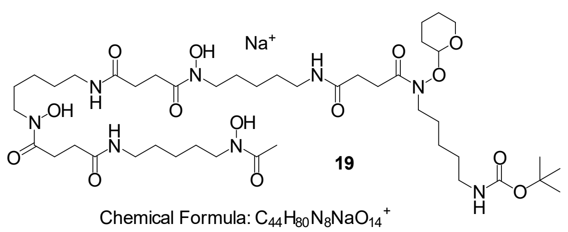

Chemical Formula: $C_{44}H_{80}N_8NaO_{14}^+$
Exact Mass: 967,5686

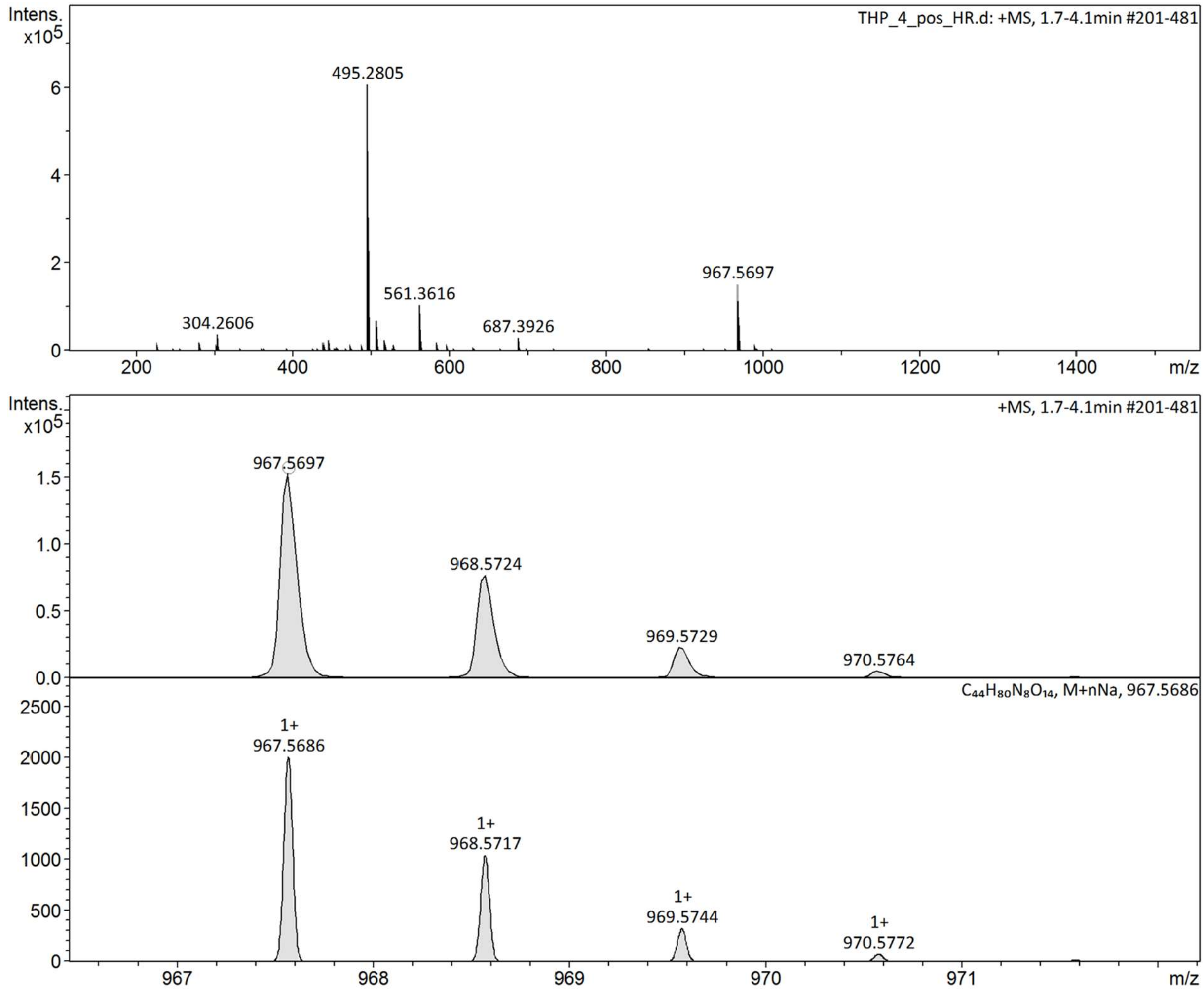

Figure S9: HR-ESI mass spectrum of **19**

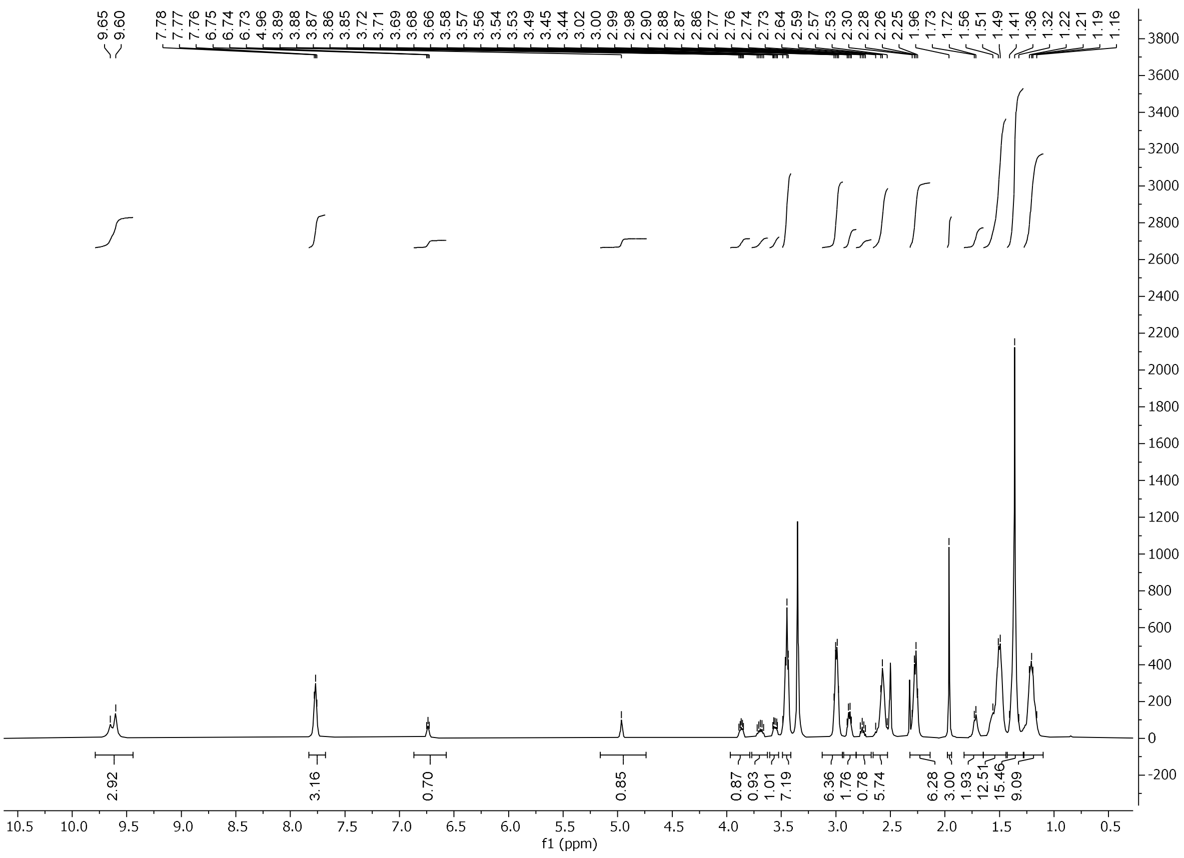

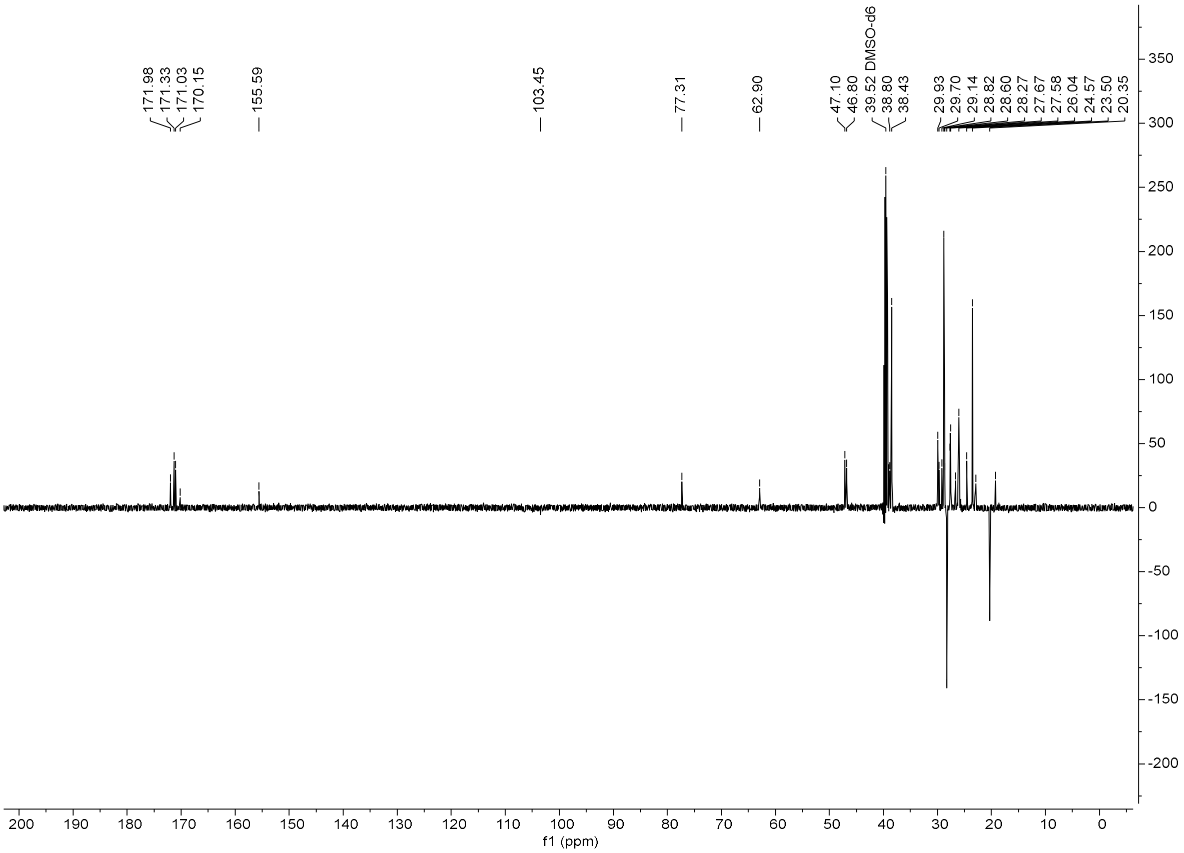

Figure S10: ^{1}H-NMR and ^{13}C-NMR of **19**

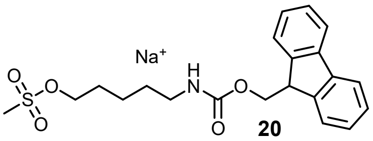

20

Chemical Formula: $C_{21}H_{25}NNaO_5S^+$
Exact Mass: 426,1346

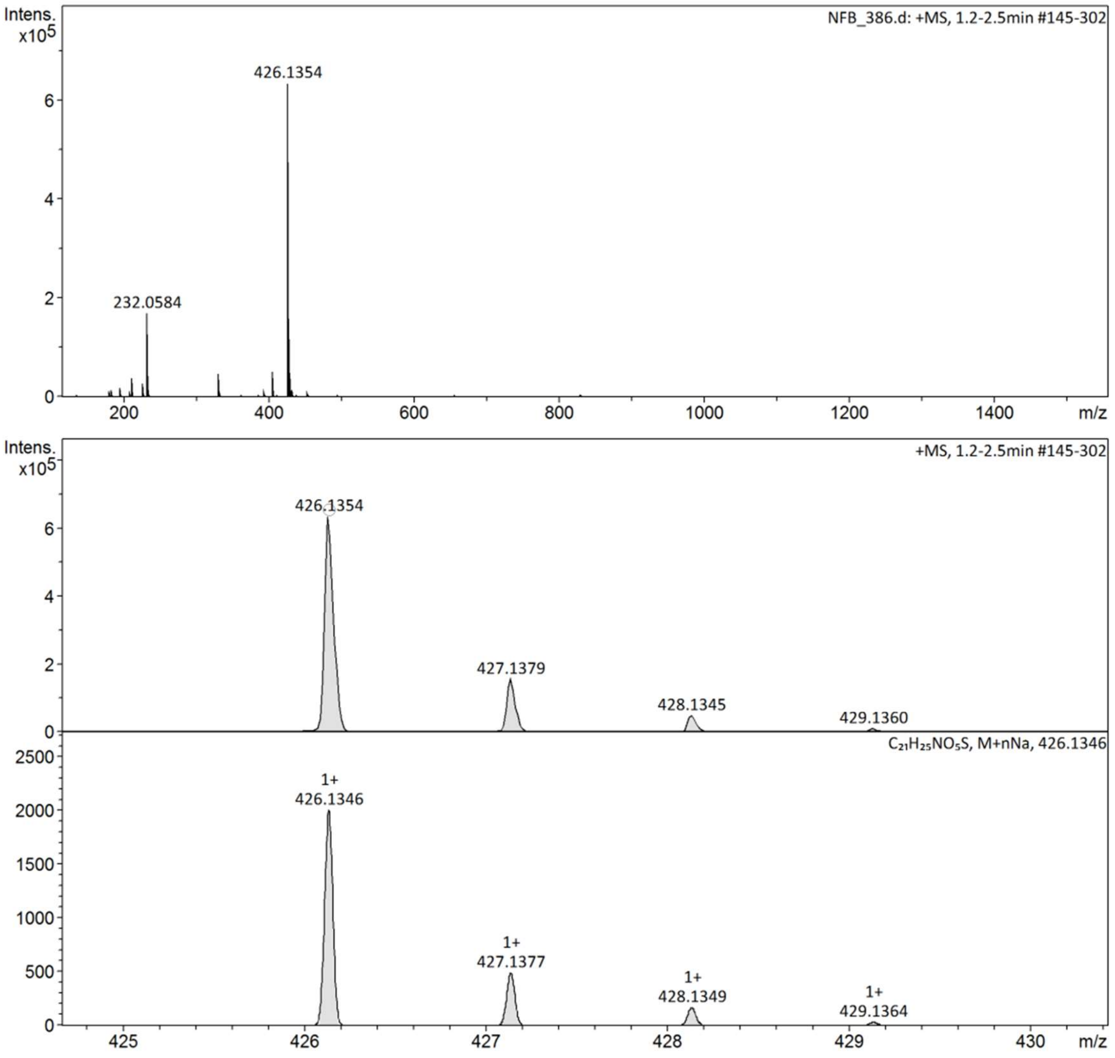

Figure S11: HR-ESI mass spectrum of **20**

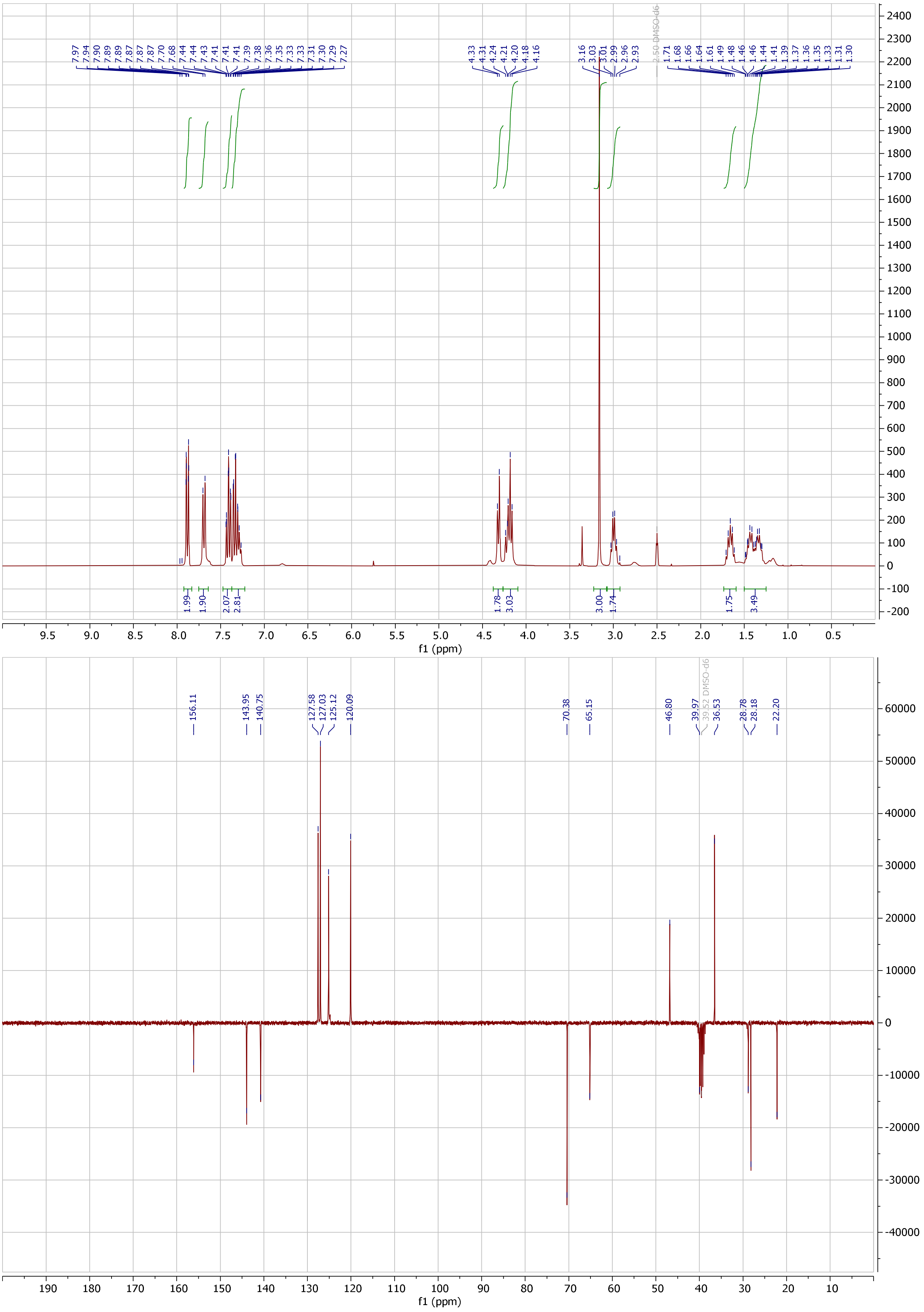

Figure S12: ^{1}H-NMR and ^{13}C-NMR of **20**

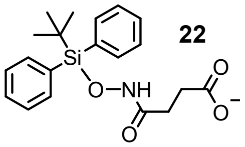

22

Chemical Formula: $C_{20}H_{24}NO_4Si^-$
Exact Mass: 370,1480

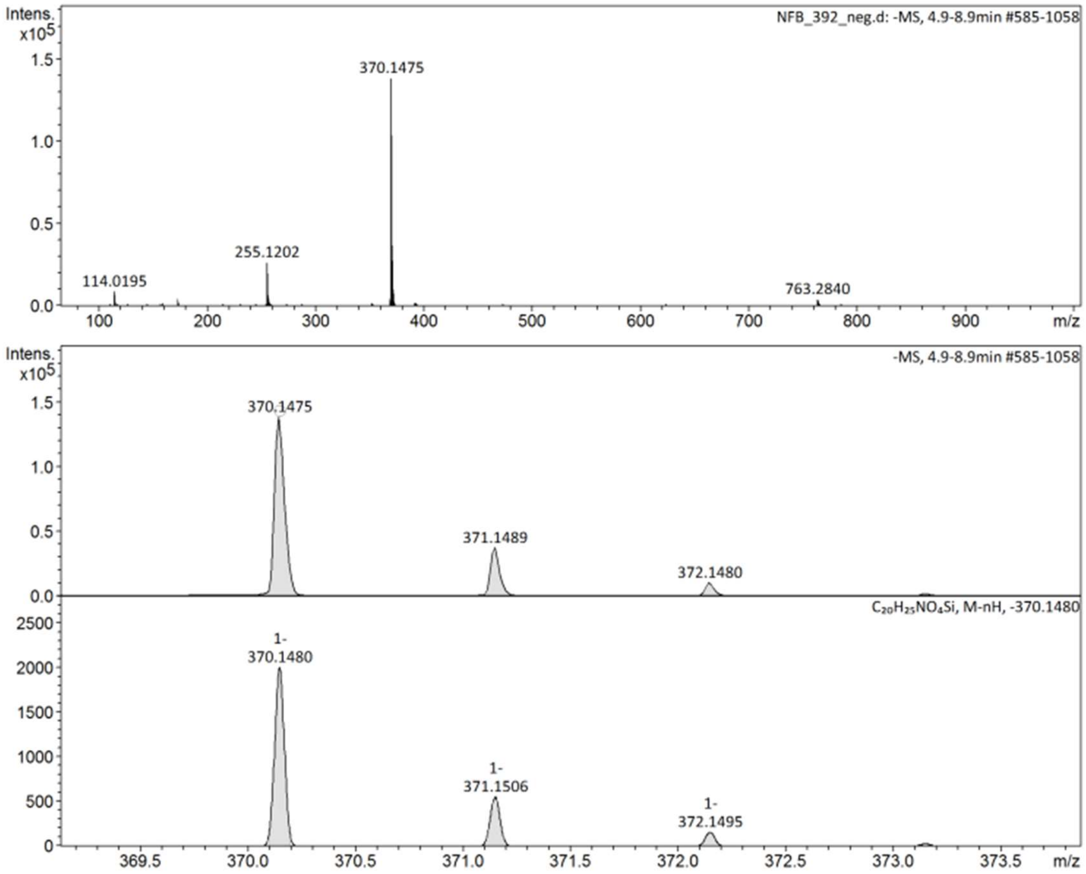

Figure S13: HR-ESI mass spectrum of **22**

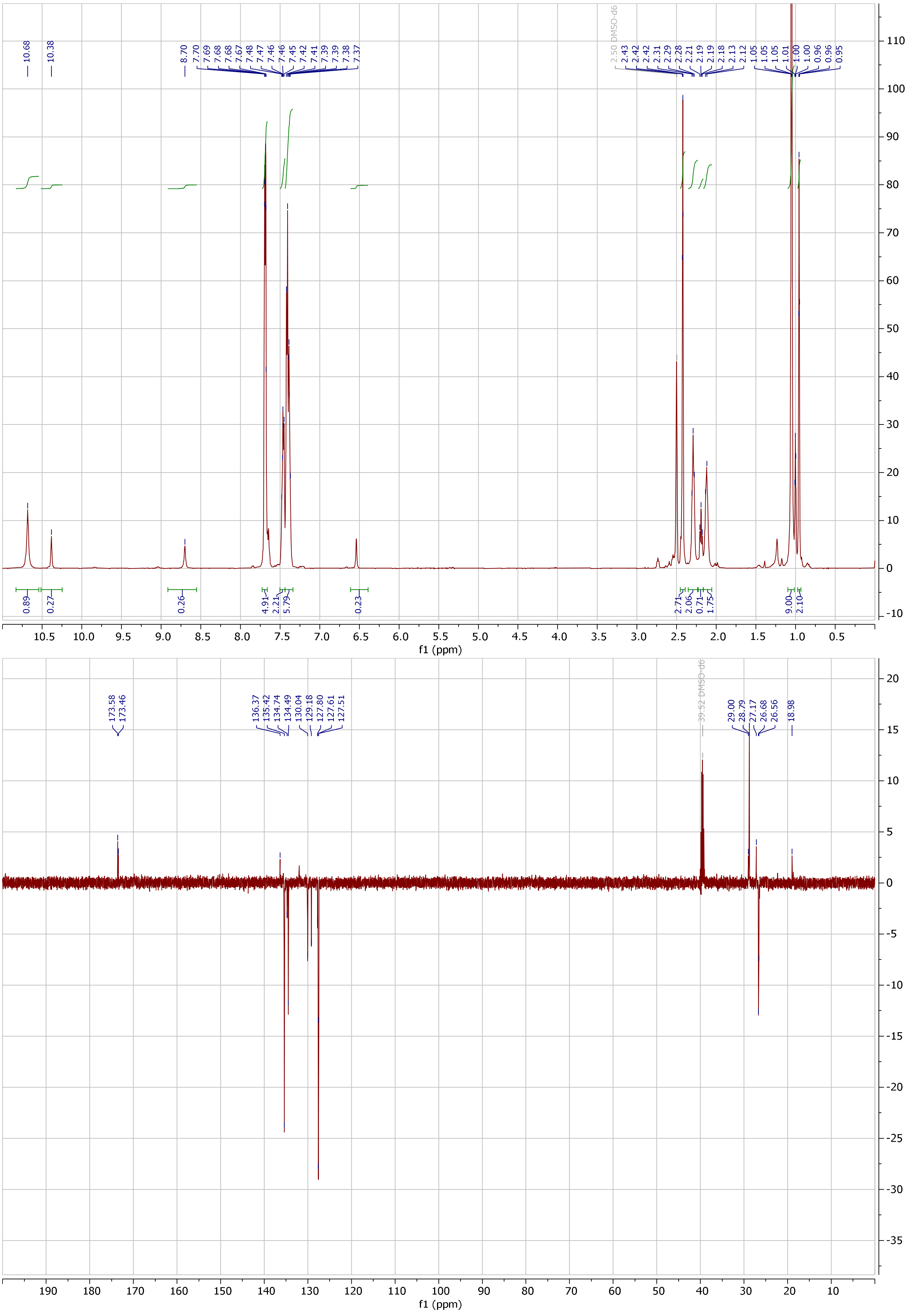

Figure S14: ^{1}H-NMR and ^{13}C-NMR of **22**

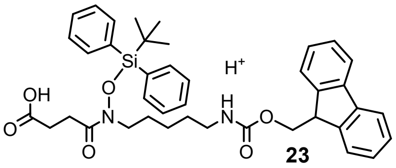

Chemical Formula: $C_{40}H_{47}N_2O_6Si^+$
Exact Mass: 679,3198

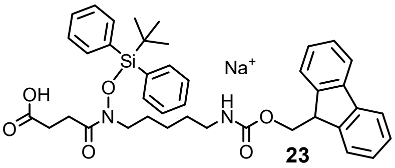

Chemical Formula: $C_{40}H_{46}N_2NaO_6Si^+$
Exact Mass: 701,3017

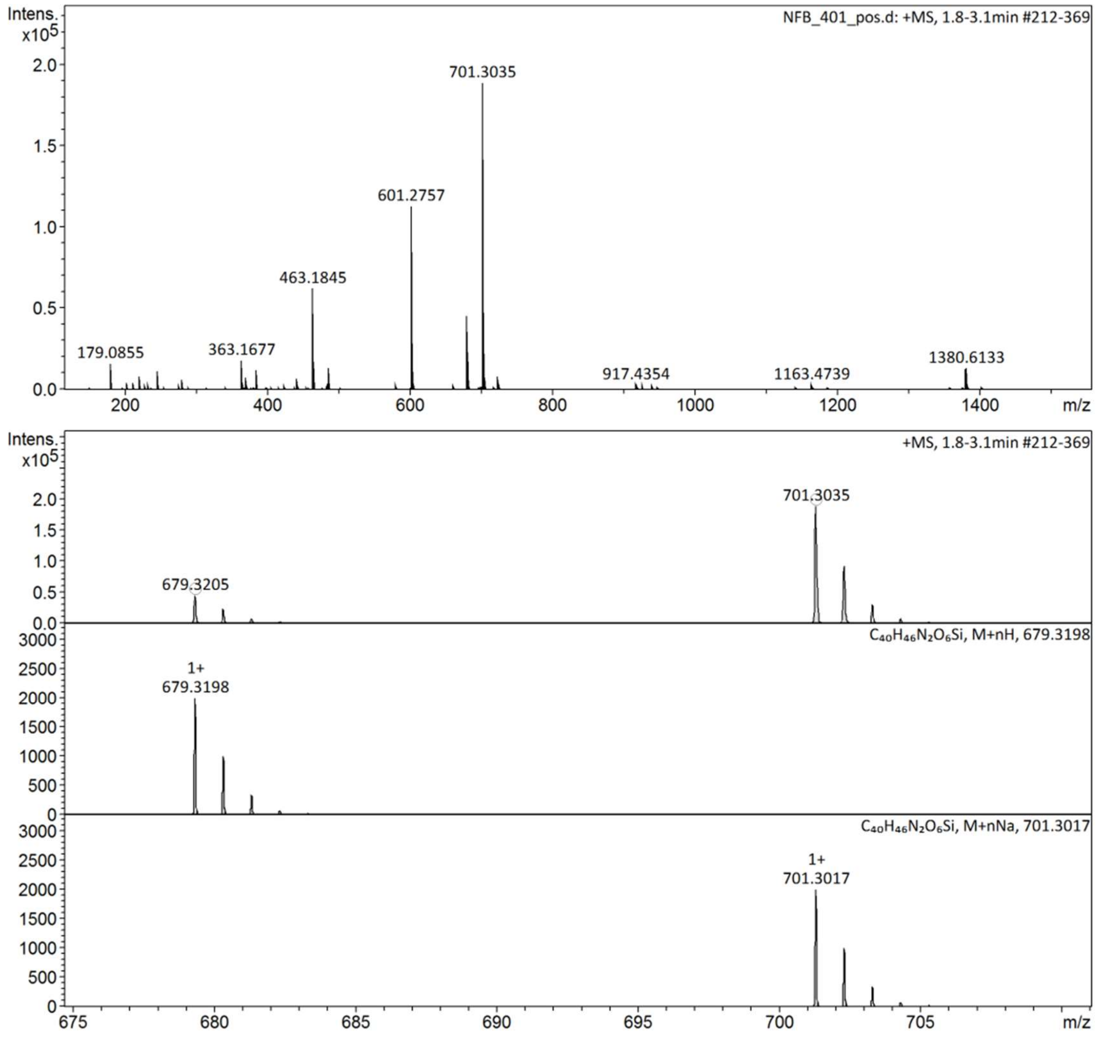

Figure S15: HR-ESI mass spectrum of **23**

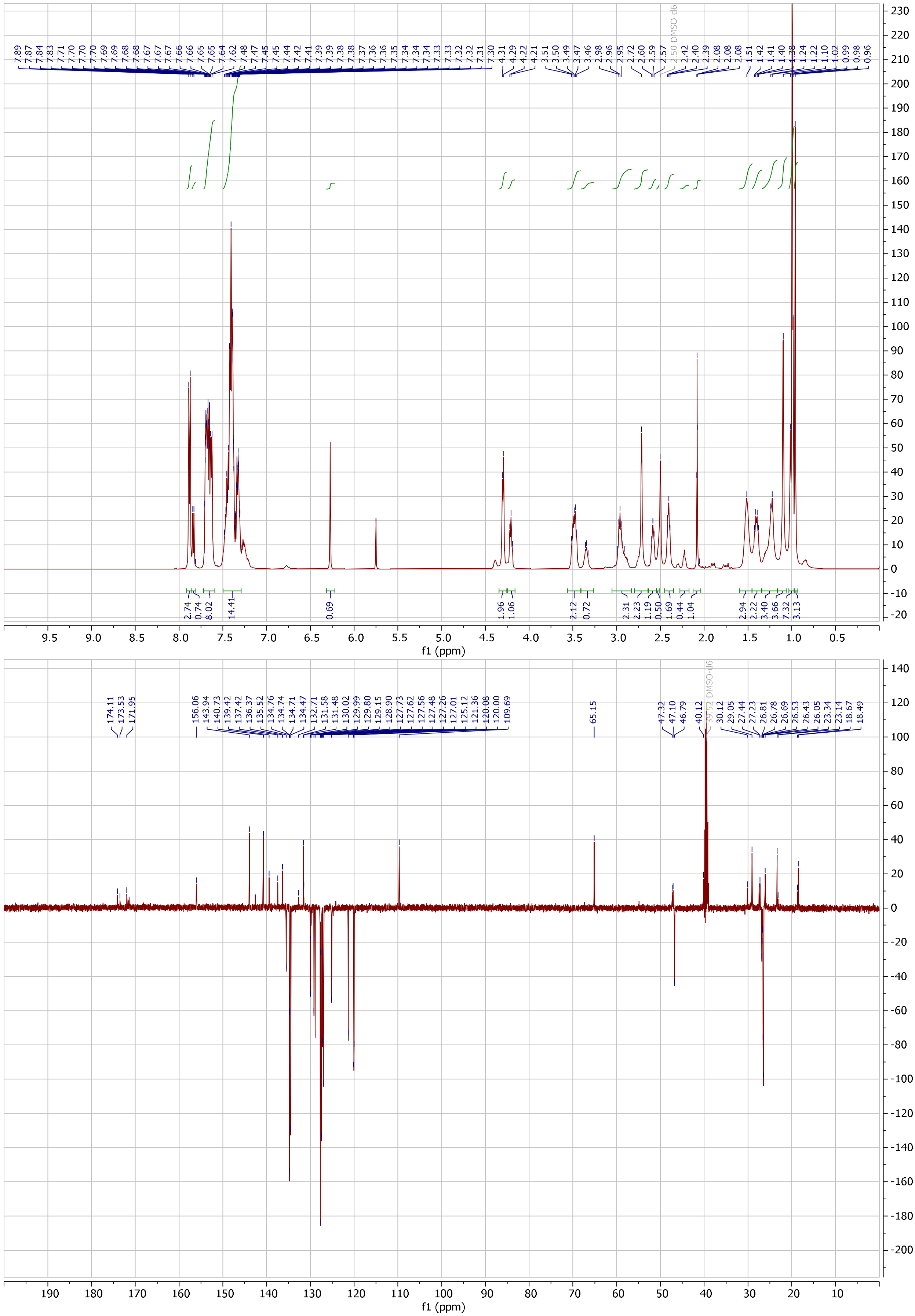

Figure S16: ^{1}H-NMR and ^{13}C-NMR of **23**

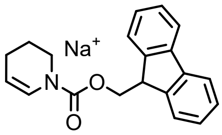

25

Chemical Formula: $C_{20}H_{19}NO_2Na^+$
Exact Mass: 328,1308

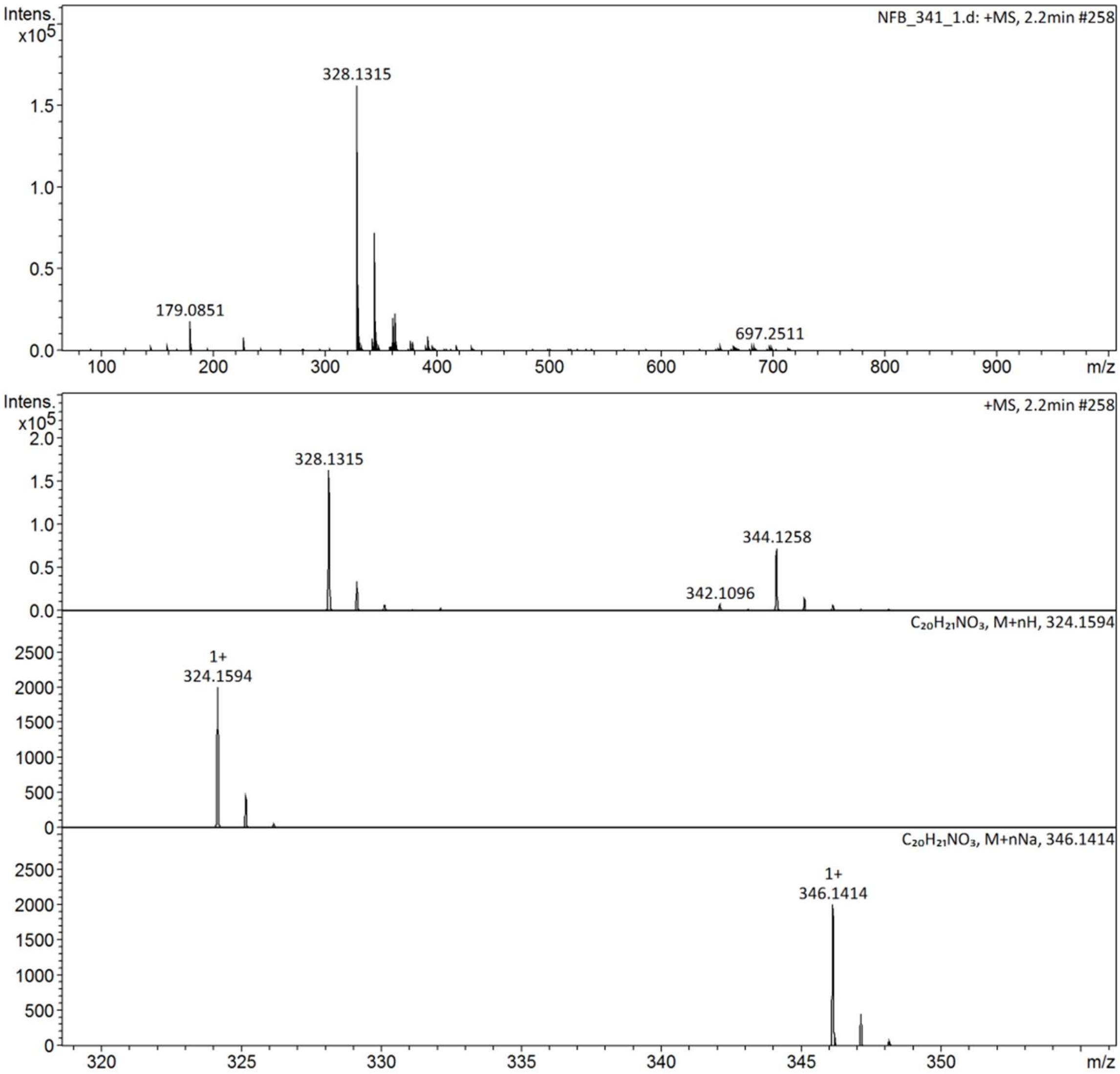

Figure S17: HR-ESI mass spectrum of **25**

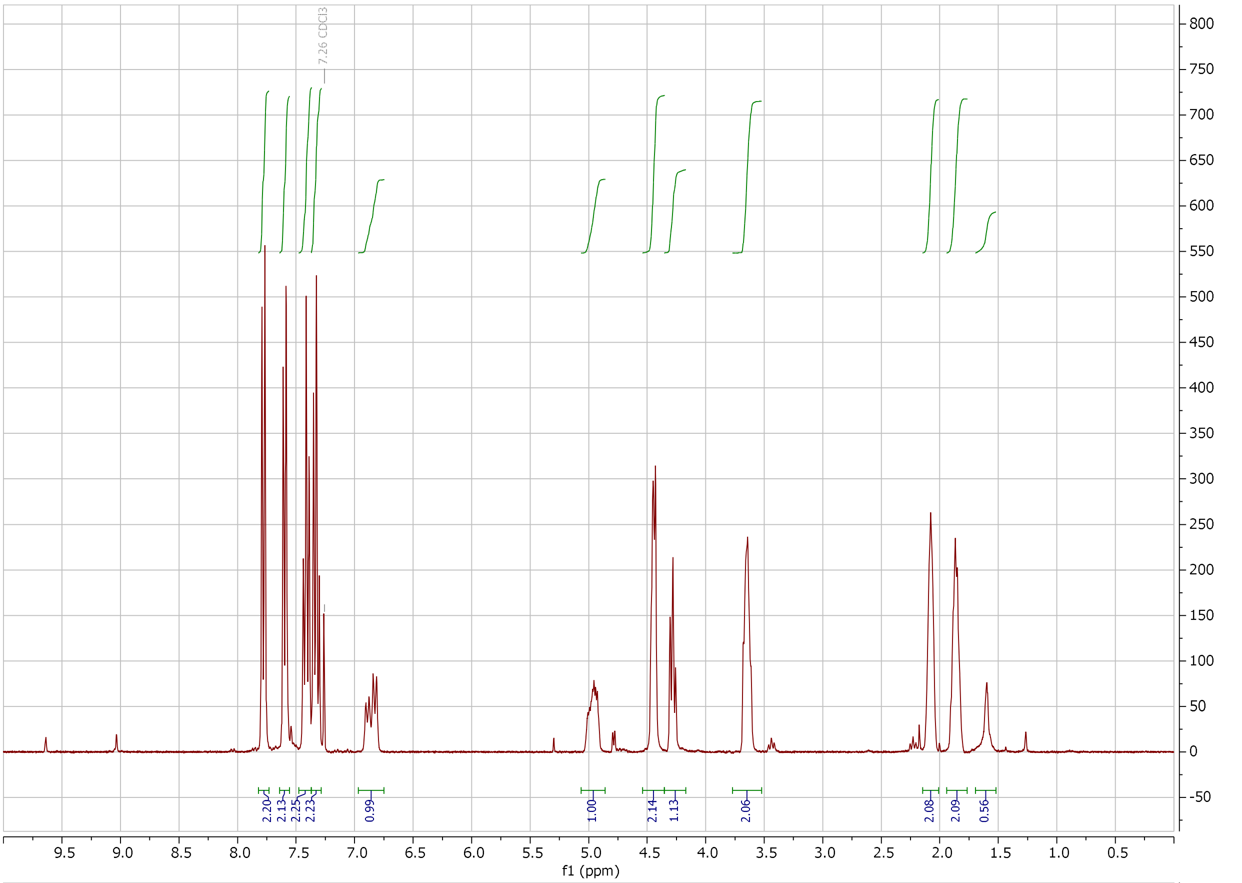

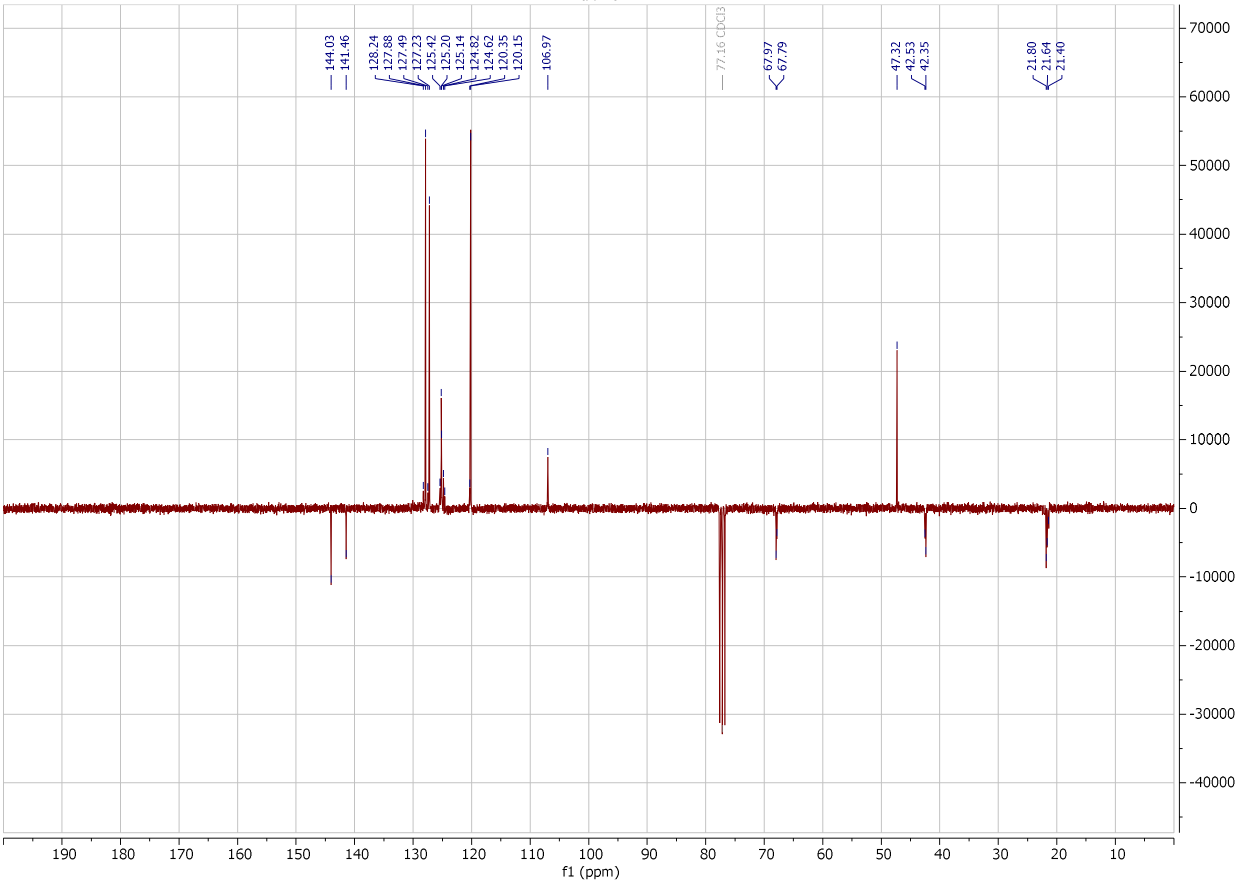

Figure S18: ^{1}H-NMR and ^{13}C-NMR of **25**

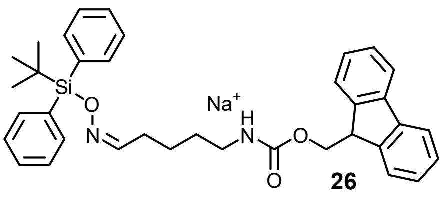

Chemical Formula: $C_{36}H_{40}N_2NaO_3Si^+$
Exact Mass: 599,2700

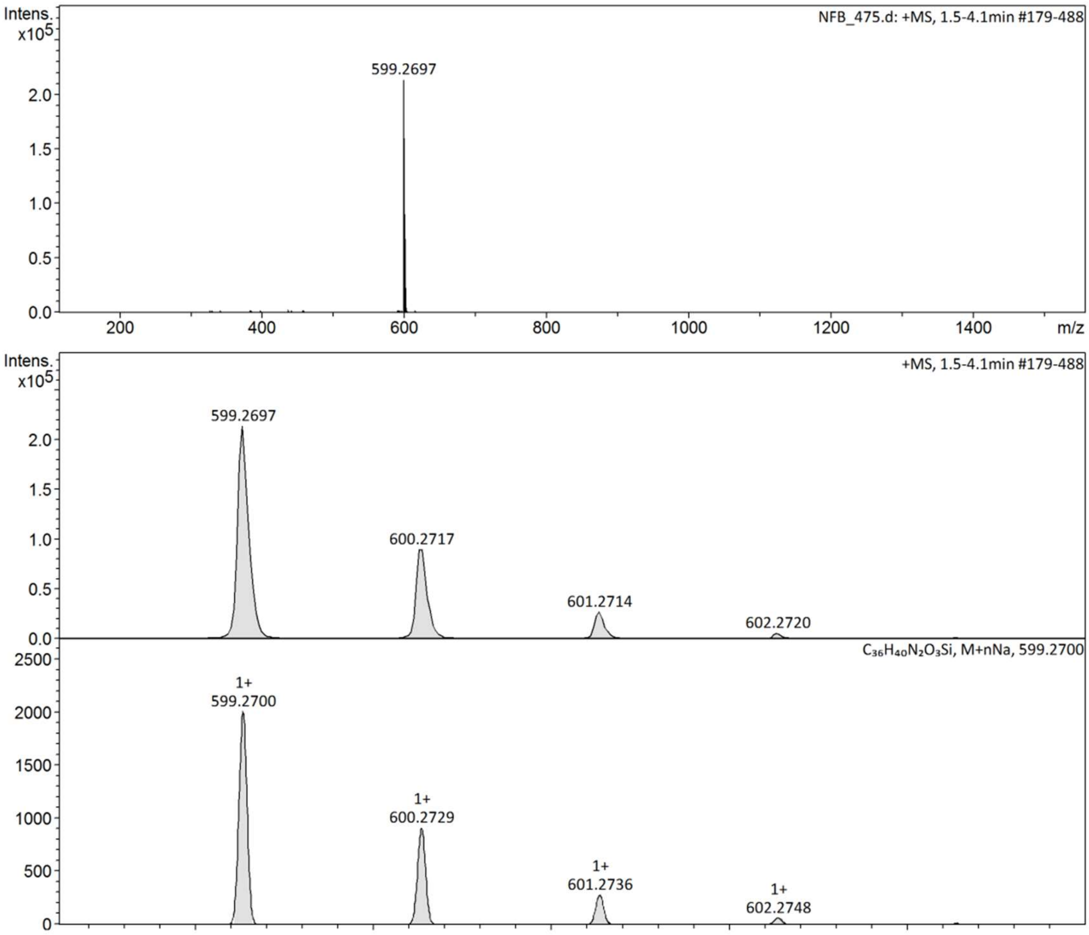

Figure S19: HR-ESI mass spectrum of **26**

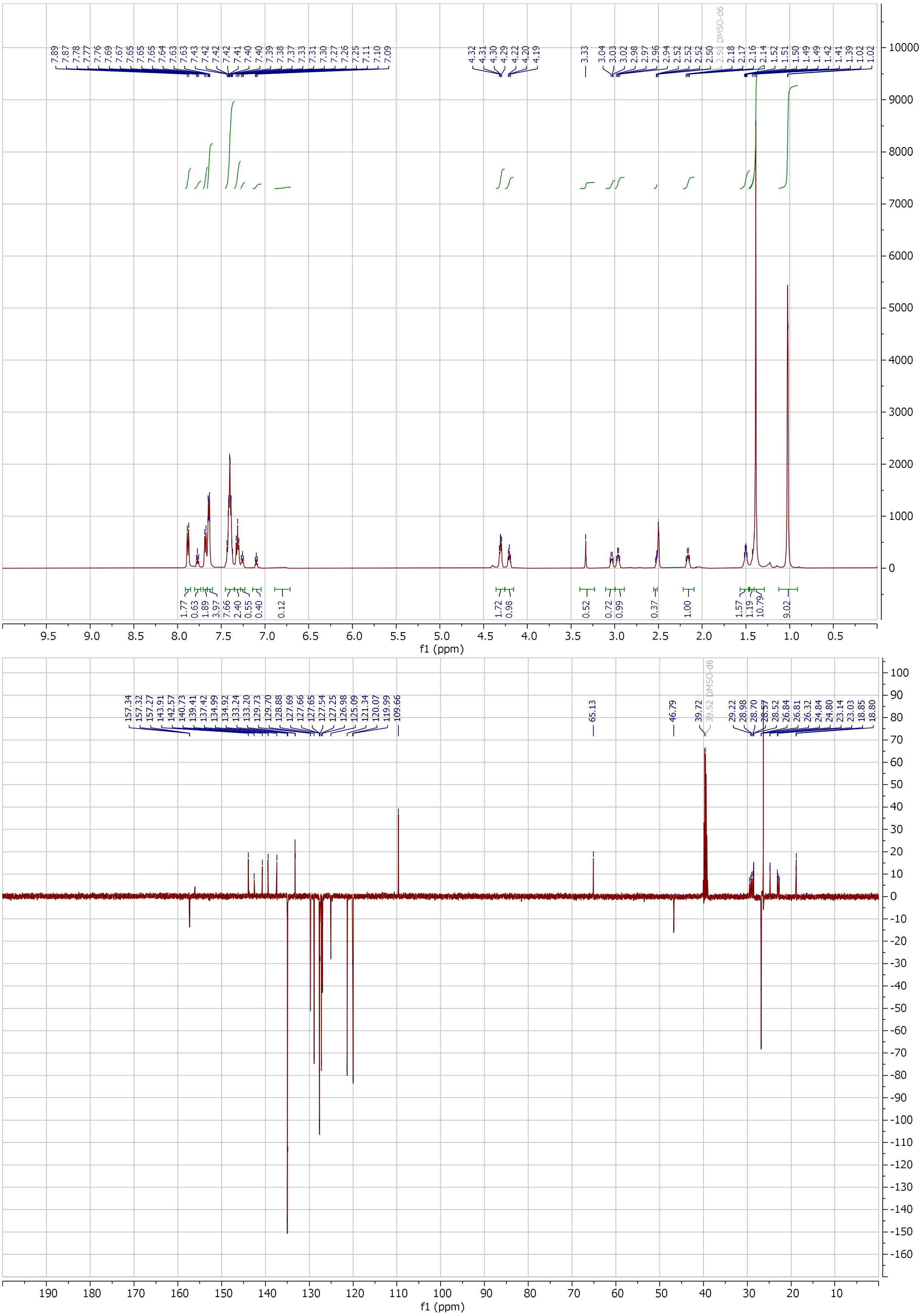

Figure S20: ^{1}H-NMR and ^{13}C-NMR of **26**

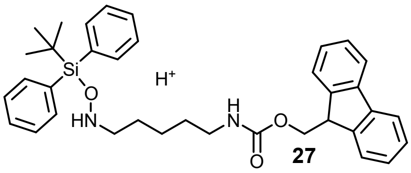

Chemical Formula: $C_{36}H_{43}N_2O_3Si^+$
Exact Mass: 579,3037

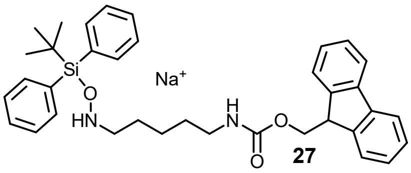

Chemical Formula: $C_{36}H_{42}N_2NaO_3Si^+$
Exact Mass: 601,2857

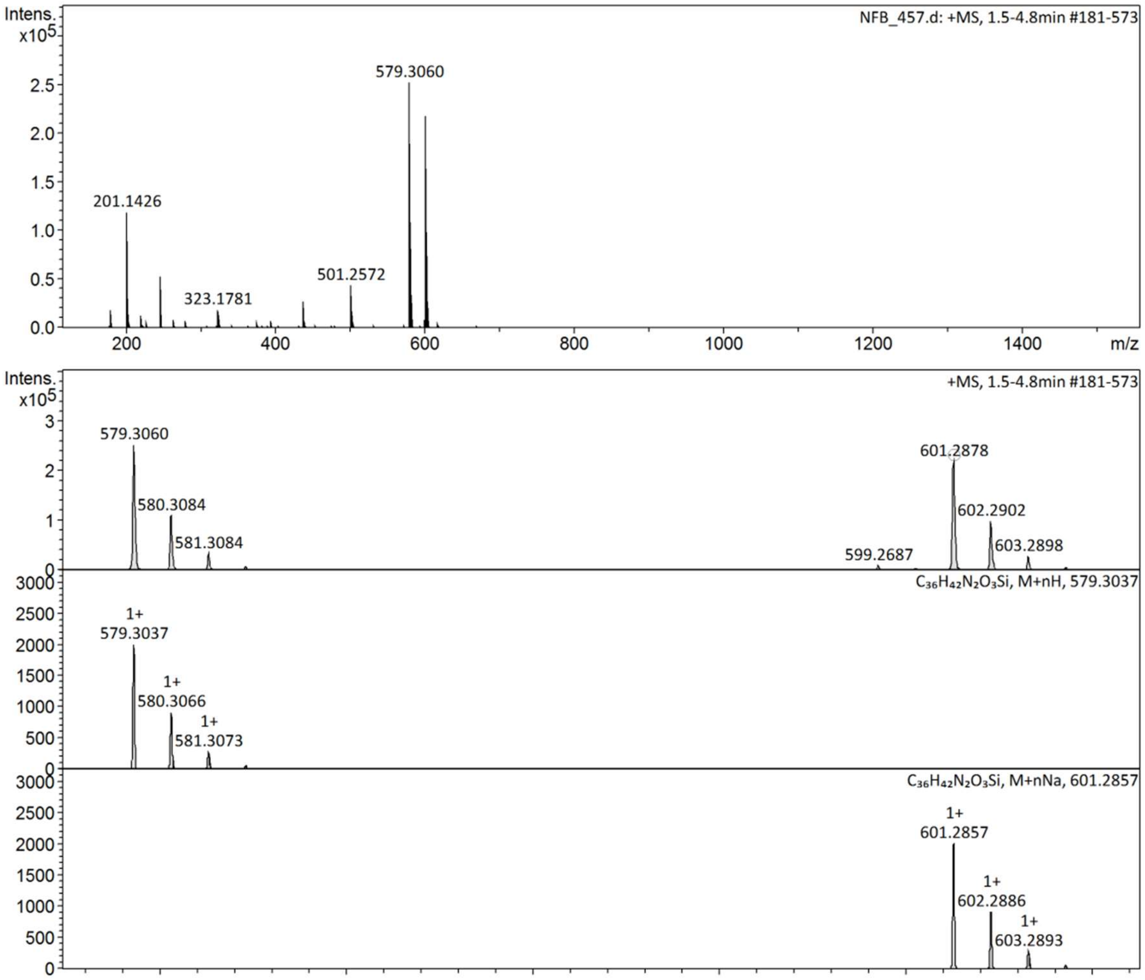

Figure S21: HR-ESI mass spectrum of **27**

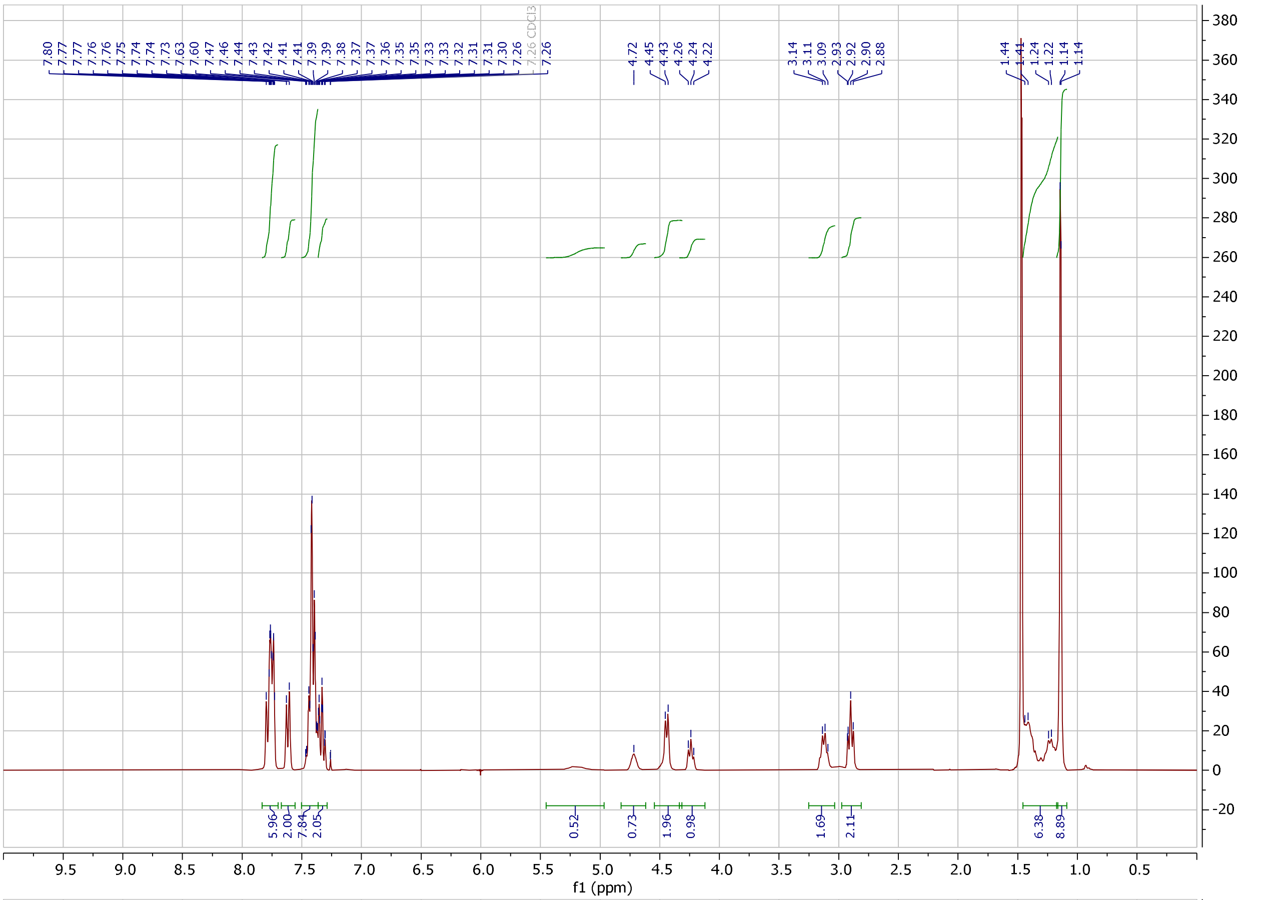

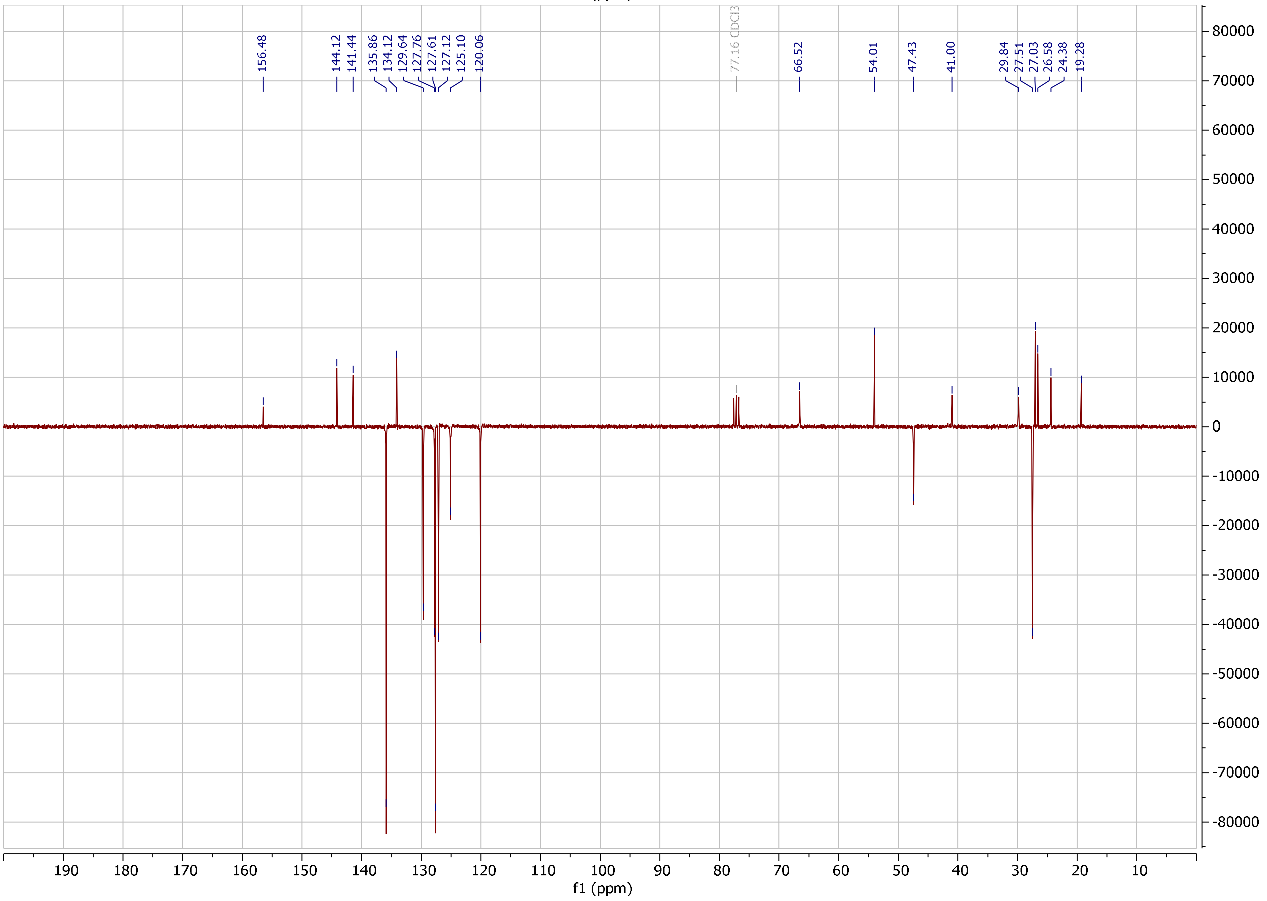

Figure S22: ^{1}H-NMR and ^{13}C-NMR of **27**

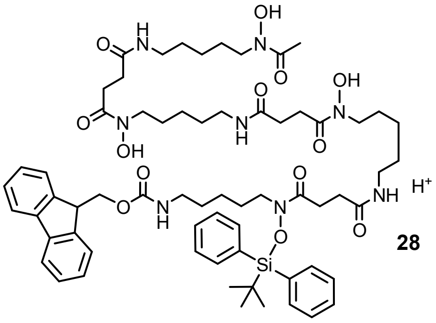

Chemical Formula: $C_{65}H_{93}N_8O_{13}Si^+$
Exact Mass: 1221,6626

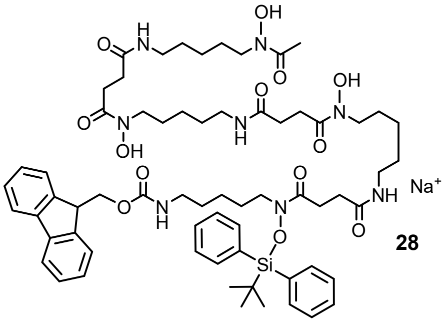

Chemical Formula: $C_{65}H_{92}N_8NaO_{13}Si^+$
Exact Mass: 1243,6445

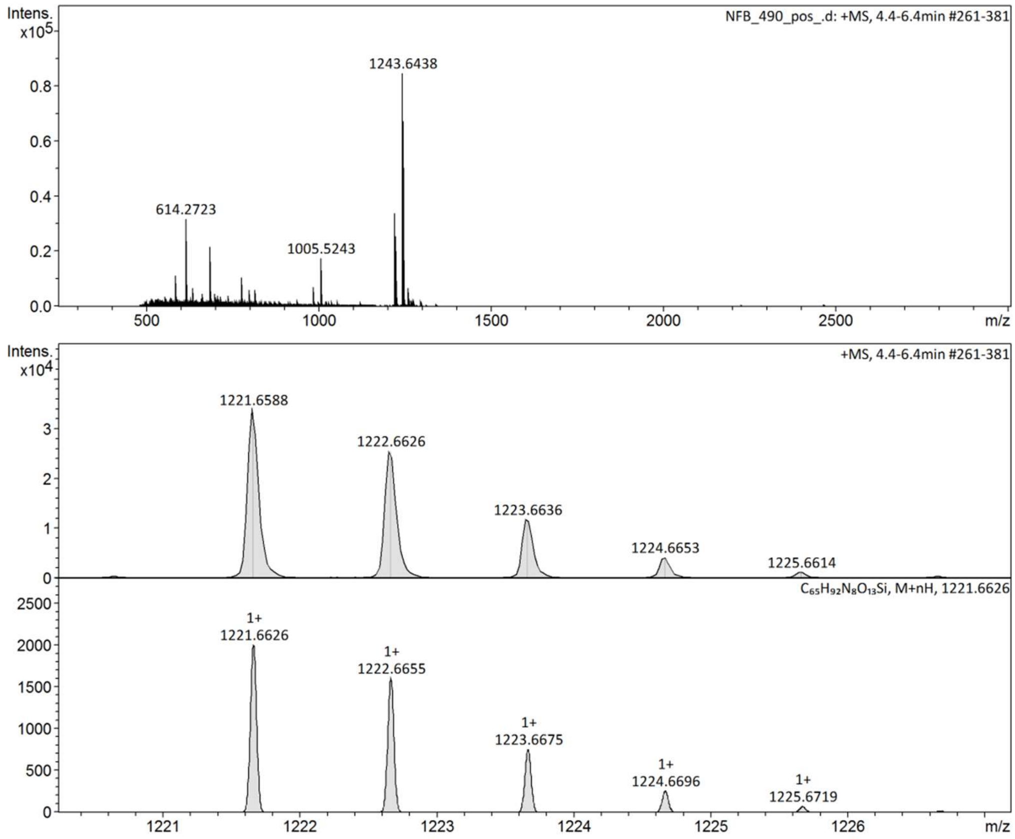

Figure S23: HR-ESI mass spectrum of **28**

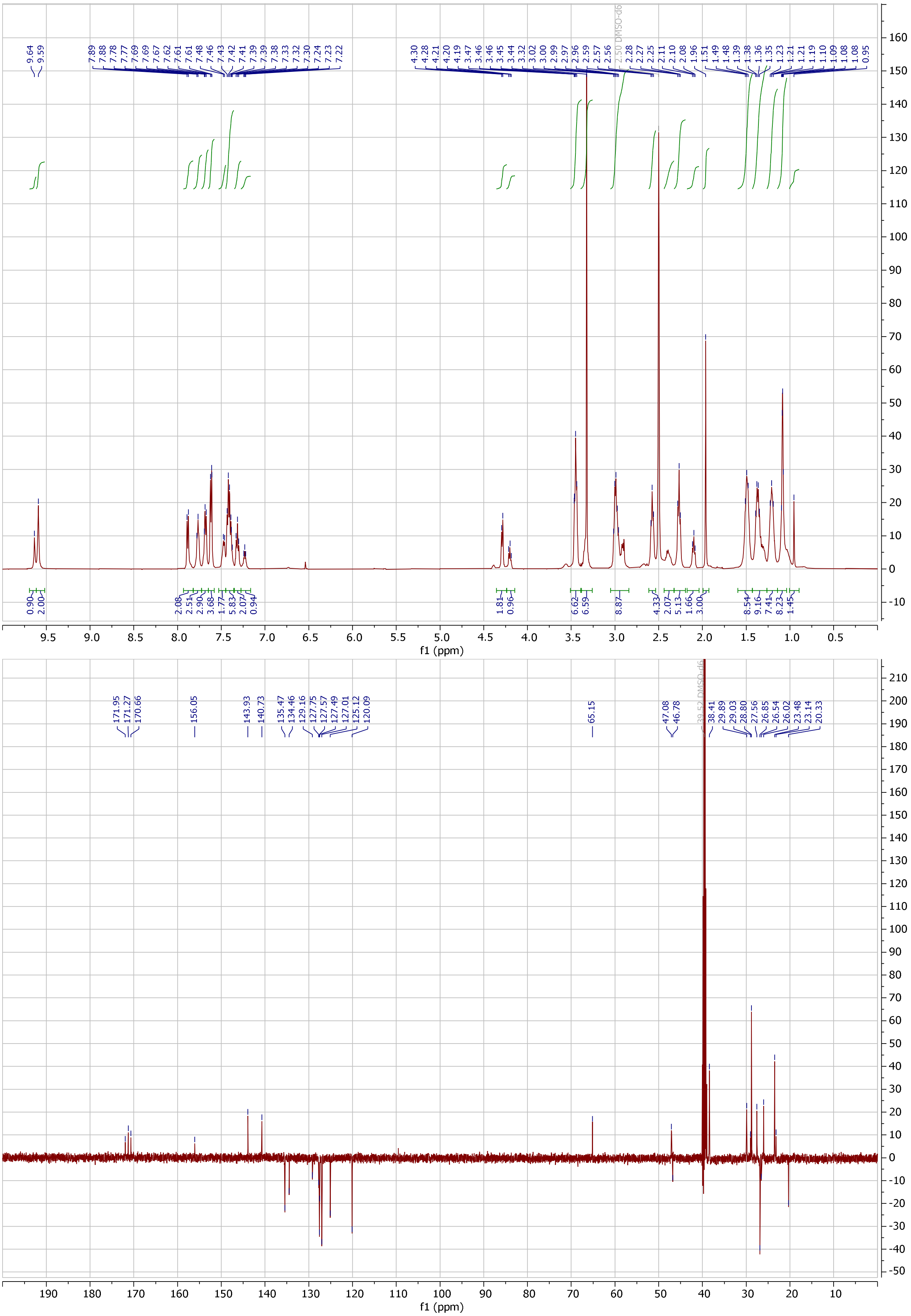

Figure S24: ^{1}H-NMR and ^{13}C-NMR of **28**

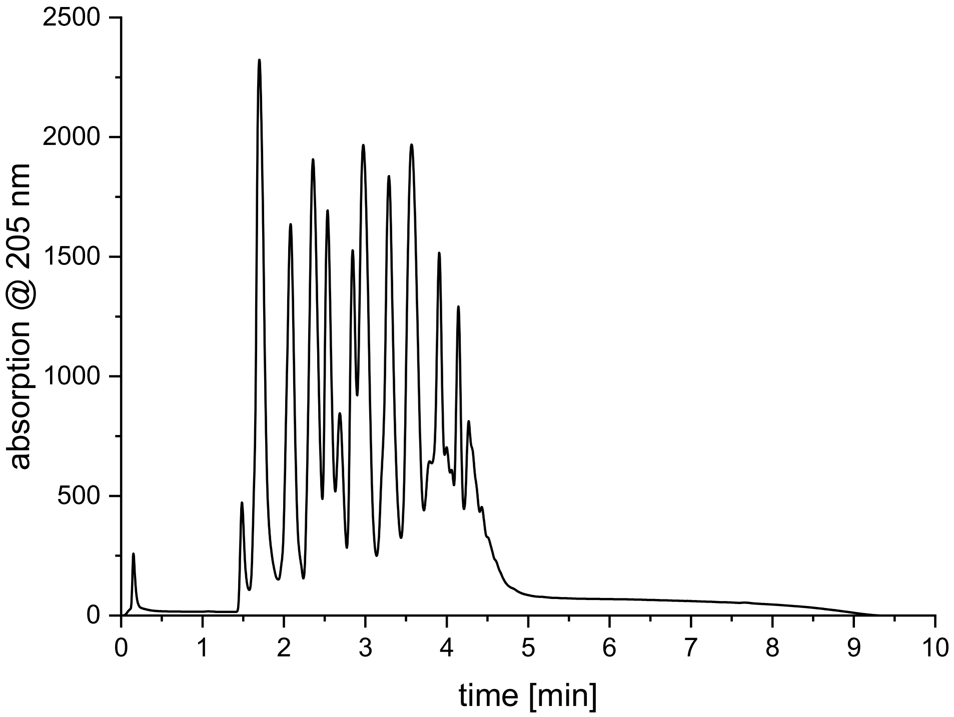

Figure S25: Analytical HPLC chromatogram of the deprotection reaction of the *tert*-butyl (^{t}Bu) and *tert*-butoxycarbonyl (Boc) protected intermediate **11** to DFO* amine **1** using 97% TFA (conditions: Chromolith performance RP-18e, gradient: 10% – 100% MeCN + 0.1% TFA in 10 minutes). The peak at 3.6 min corresponds to product.
